# Myeloid-like tumor hybrid cells in bone marrow promote progression of prostate cancer bone metastasis

**DOI:** 10.1186/s13045-023-01442-4

**Published:** 2023-05-03

**Authors:** Xinyu Ye, Xin Huang, Xing Fu, Xiao Zhang, Risheng Lin, Wen Zhang, Jian Zhang, Yi Lu

**Affiliations:** School of Medicine, Southern University of Science and Technology, No. 1088 Xue Yuan Blvd, Shenzhen, 518055 Guangdong China

## Abstract

**Background:**

Bone metastasis is the leading cause of death in patients with prostate cancer (PCa) and currently has no effective treatment. Disseminated tumor cells in bone marrow often obtain new characteristics to cause therapy resistance and tumor recurrence. Thus, understanding the status of disseminated prostate cancer cells in bone marrow is crucial for developing a new treatment.

**Methods:**

We analyzed the transcriptome of disseminated tumor cells from a single cell RNA-sequencing data of PCa bone metastases. We built a bone metastasis model through caudal artery injection of tumor cells, and sorted the tumor hybrid cells by flow cytometry. We performed multi-omics analysis, including transcriptomic, proteomic and phosphoproteomic analysis, to compare the difference between the tumor hybrid cells and parental cells. In vivo experiments were performed to analyze the tumor growth rate, metastatic and tumorigenic potential, drug and radiation sensitivity in hybrid cells. Single cell RNA-sequencing and CyTOF were performed to analyze the impact of hybrid cells on tumor microenvironment.

**Results:**

Here, we identified a unique cluster of cancer cells in PCa bone metastases, which expressed myeloid cell markers and showed a significant change in pathways related to immune regulation and tumor progression. We found that cell fusion between disseminated tumor cells and bone marrow cells can be source of these myeloid-like tumor cells. Multi-omics showed the pathways related to cell adhesion and proliferation, such as focal adhesion, tight junction, DNA replication, and cell cycle, were most significantly changed in these hybrid cells. In vivo experiment showed hybrid cells had a significantly increased proliferative rate, and metastatic potential. Single cell RNA-sequencing and CyTOF showed tumor-associated neutrophils/monocytes/macrophages were highly enriched in hybrid cells-induced tumor microenvironment with a higher immunosuppressive capacity. Otherwise, the hybrid cells showed an enhanced EMT phenotype with higher tumorigenicity, and were resistant to docetaxel and ferroptosis, but sensitive to radiotherapy.

**Conclusion:**

Taken together, our data demonstrate that spontaneous cell fusion in bone marrow can generate myeloid-like tumor hybrid cells that promote the progression of bone metastasis, and these unique population of disseminated tumor cells can provide a potential therapeutic target for PCa bone metastasis.

**Supplementary Information:**

The online version contains supplementary material available at 10.1186/s13045-023-01442-4.

## Introduction

Prostate cancer (PCa) is the second most prevalent malignancy in men worldwide [1, 2]. The treatments for early-stage PCa include surgery, chemotherapy, radiotherapy, and castration therapy [3]. Although most patients with PCa are diagnosed in the early stage and have a good prognosis after treatment, there remain many patients who are at an advanced stage when they are diagnosed [4]. Distant metastasis occurs in most cases of advanced PCa and is the leading cause of death in PCa. Bone is the most common metastatic site in PCa [5]. Bone metastasis can cause severe bone pain, pathological bone fracture, and hypercalcemia, all of which seriously lower the living quality and overall survival of patients with PCa. The median survival of patients with PCa with bone metastasis is less than 3 years, and the average 5-year survival rate is only 3% [6]. Currently, chemotherapy, androgen deprivation therapy and immunotherapy are the main treatments for PCa metastasis. However, most patients will eventually develop therapy resistance, which is the main cause of treatment failure in PCa [7].

The development of bone metastasis is a multi-step process, involving circulation of tumor cells into the bone marrow before entering into dormancy status, followed by reactivation and proliferation in the bone marrow, and reconstruction of the bone [8]. Unfortunately, more than half of patients with PCa show bone metastases when they are initially diagnosed, and it can take more than 10 years to form clinical overt metastatic recurrence due to the presence of dormant disseminated tumor cells (DTCs) in the bone marrow. The mechanisms that cause dormancy in DTCs include autophagy in tumor cells, signals in stem cell niches, and interaction with extracellular matrix or immune cells [9, 10]. The role of disseminated tumor cells in tumor progression has gained increasing attention recently, particularly because they can remain in a dormant state to escape from chemotherapy and radiation [11]. Numerous studies have demonstrated that DTCs can act as a prognostic marker for relapse and represent a potential target for cancer therapy [12–15].

Compared with primary tumor cells, DTCs in the bone marrow often experience a significant evolution due to obtaining new characteristics during the progression of bone metastasis [16]. For example, most DTCs in the bone marrow show properties of epithelial mesenchymal transition (EMT) with up-regulation of N-cadherin and down-regulation of E-cadherin [17], as well as an osteoblast-like/osteoclast-like phenotype to help them survive in the bone marrow [18, 19]. Thus, understanding the status of disseminated PCa cells in the bone marrow microenvironment is urgently needed for the development of new treatments for bone metastasis. In this study, we identified a cluster of disseminated PCa cells expressing myeloid-cell markers in PCa bone metastasis. We found that cell fusion is one of the sources of these myeloid-like cells. The in vivo animal experiments show that these myeloid-like hybrid tumor cells have higher tumorigenic and metastatic potential, can induce a more immunosuppressive microenvironment, and cause drug resistance. Our findings suggest that cell fusion can generate myeloid-like tumor cells that promote bone metastasis in PCa.

## Methods

### Cell culture

The mouse PCa cell line RM1 was purchased from American Type Culture Collection (ATCC) and was tested against mycoplasma contamination. RM1 cells and hybrid tumor cells were cultured in DMEM containing 10% FBS and 1% penicillin/streptomycin and maintained in a 37 °C incubator with 5% CO_2_.

### Single-cell RNA-seq data analysis of PCa patient bone metastasis samples

Previously published scRNA-seq data of bone metastases from patients with PCa were downloaded from the GEO Datasets (GSE143791) [20]. The gene count matrices from GEO were converted to Seurat objects by Seurat R package (version 4.1.1) and used for all further analysis. Cells with a UMI > 600 were selected for further analysis. The total count of each cell was normalized to 10,000. The number of principal components (PCs) was adjusted to 14 to generate cell clusters that were then exhibited using Uniform Manifold Approximation and Projection (UMAP). The cell clusters were annotated based on the information downloaded from GEO (GSE143791) and cell markers from CellMarker (http://xteam.xbio.top/CellMarker/). Considering that the effect of tumor heterogeneity is much greater than the batch effect, to preserve the difference between tumor cells from different samples, we did not perform batch effect adjustment during scRNA-seq dataset integration. Differential expression analysis between selected clusters was performed using the “FindMarksers” method. GO, KEGG, and GSEA enrichment analyses of differentially expressed genes were performed using the “clusterProfiler” R package (v4.2.2).

### Sorting of hybrid tumor cells from the bone marrow of mice with bone metastasis

RM1 (mcherry-nls-luciferase) was generated by lentiviral transduction using pRlenti-based lentiviruses. Briefly, 1 × 10^5^ RM1 (mcherry-nls-luciferase) was injected into EGFP C57/BL6J mice through the caudal artery to build a bone metastasis model. The mice were euthanized when there was significant bioluminescence in the bone area, and then the bone marrow cells were flushed out with PBS for FACS sorting. The double-positive bone marrow cells were sorted into a 6-well plate by FACS. After the proliferation of sorted double-positive cells in vitro for several passages, we sorted the single double-positive cells into 96-well plates using FACS to form single-cell colonies. The hybrid cells from single colonies were used for further experiments.

### Polymerase chain reaction (PCR)

The genomic DNA of RM1 cells, macrophages, and hybrid cells was extracted using a Genome isolation kit (TIANGEN, DP304-02) according to the manufacturer’s instructions. The EGFP primers were as follows: 5′-AAGGGCATCGACTTCAAGG-3′ (forward) and 5′-TGCTTGTCGGCCATGATATAG-3′ (reverse). The mCherry primers were as follows: 5′-GATGGTGTAGTCCTCGTTGTG-3′ (forward) and 5′-CCCCGTAATGCAGAAGAAGA-3′ (reverse). The polymerase chain reaction (PCR) was performed using KOD FX 2004 (TOYOBO) according to the manufacturer’s instructions.

### Transcriptomics analysis

The total RNA of RM1 and hybrid cells was extracted using an RNA isolation kit (Vazyme, RC112-01) according to the manufacturer’s instructions. The cDNA libraries were prepared using an Illumina TrueSeq RNA Sample Preparation Kit V. 4.1 (Illumina Inc., San Diego, CA) and sequenced using Illumina HiSeq2500 by Gene Denovo Biotechnology Co. (Guangzhou, China).

The raw data were also processed and analyzed by Gene Denovo Biotechnology Co. Quality control was performed using Fast (version 0.18.0) to remove reads containing adapters and low-quality bases. The ribosome RNA was removed according to the result of alignment using Bowtie2. The raw reads were then aligned to the mouse reference genome of Ensembl_release98. Differential expression analysis was performed using DEseq2. The genes with FDR < 0.05 and |Log_2_(fold change)|> 1 were considered differentially expressed genes (DEGs). GO and KEGG enrichment were performed to identify the main biological function of DEGs. GSEA was performed using the software GSEA and MSigDB.

### Label-free proteomic analysis

The protein samples were extracted and digested overnight. The supernatant of the digested sample was loaded onto a C18 desalting column and elution buffer. The eluents were collected and lyophilized. The lyophilized powder was resolved in 10 μL of mobile phase solution A (100% water, 0.1% formic acid), centrifuged, and introduced onto a C18 Nano-Trap column. Separated peptides were analyzed by Orbitrap Exploris 480 along with FAIMS (Thermo Fisher) and Nanospray FlexTM (ESI). The acquired spectra were searched against the Mus_musculus_uniprot_2020_7_2. fasta (86,555 sequences) database and filtered by the Proteome Discoverer 2.4 (PD 2.4, Thermo). Peptide spectrum matches (PSMs) with credibility > 99% were identified as credible PSMs, and the identified protein containing at least one unique peptide was considered a credible protein. The identified PSMs and protein were retained and performed with an FDR of ≤ 1%. Differentially expressed proteins (DEPs) were used for volcanic map analysis, and enrichment analysis of GO and KEGG.

### Phosphoproteomic analysis

The samples were digested, loaded onto a C18 cartridge, and dried by vacuum centrifugation. All of the phosphopeptides were enriched by PHOS-Select™ Iron Affinity Gel (Sigma, P9740). The bound peptides were then desalted by spin columns (Thermo Fisher, 89,852). Shotgun proteomics analyses were performed using an EASY-nLCTM 1200 UHPLC system (Thermo Fisher) coupled with an Orbitrap Q Exactive HF-X mass spectrometer (Thermo Fisher) measured in a data-dependent acquisition (DDA) mode. Peptides were eluted on a homemade column (15 cm × 150 µm, 1.9 µm) with a 120-min gradient starting at 5–10% buffer B (0.1% formic acid in 80% ACN), followed by a stepwise increase to 10–40% in 105 min, 40–50% in 5 min, 50–90% in 3 min, and 90–100% in 5 min in buffer A (0.1% formic acid in H_2_O) at a flow rate of 600 nL/min. For protein identification, proteins with at least one unique peptide were identified at a false-discovery rate (FDR) < 1% on peptide and protein levels. Proteins containing similar peptides that could not be distinguished on the basis of MS/MS analysis were grouped separately as protein groups. Precursor quantification based on intensity was used for label-free quantification. Significantly up- or down-regulated quantified proteins were determined by Mann–Whitney test, and the significant ratios, defined as *P* ≤ 0.05 and fold change (FC) ≥ 2.0 (FC ≤ 0.50), were used to filter the differentially expressed proteins (DEPs).

### Untargeted metabolomics analysis

We performed untargeted metabolomics using high-throughput liquid chromatography with untargeted high-resolution mass spectrometry. In brief, 100 μL sample was added to a 1.5-mL EP tube with 300 μL methanol and 20 μL internal standard, vortexed for 30 s, and sonicated in an ice bath for 10 min, before leaving to stand at − 20 °C for 1 h. The solution was centrifuged at 13,000 rpm for 15 min, and the supernatant was added to a chromatographic sample bottle. Next, 20 μL supernatant from each sample was mixed to be used as QC samples. Chromatographic separation was performed on a UPLC BEH Amide. The mass spectra data were collected using the AB 5600 Triple TOF system. The raw data were converted to mzXML format files using ProteoWizard software. Then, the XCMS method was used to obtain the m/z, retention time, and intensity of each peak from the mzXML data. Meanwhile, metabolites were identified according to a previous Mass Database. Data normalization was performed using the peak area normalization method. KEGG enrichment analysis of differential metabolites was performed using the “clusterProfiler” R package (v4.2.2).

### Animal experiments

All animal experiments and procedures were approved and performed in accordance with the Animal Experimentation Ethics Committee of Southern University of Science and Technology. RM1 (5 × 10^5^) and hybrid tumor cells (5 × 10^5^) were suspended in 100 μL PBS. Tumor cells were injected subcutaneously into the flank of 6- to 8-week-old C57BL/6 male mice. Tumor volumes were measured every 3 days, and the volume was calculated as follows: length × width^2^/2. Mice were euthanized when the tumor volume exceeded 2000 mm^3^ [21]. For the bone metastasis model, the mice were injected with 1 × 10^5^ tumor cells through the caudal artery [22]. The growth of bone metastases was monitored by the bioluminescence of tumor cells using a living animal imaging system (PerkinElmer, IVIS Spectrum). A CT scanner was used to detect bone destruction. For the lung metastasis model, the mice were injected with 1 × 10^5^ tumor cells through the tail vein. The growth of lung metastases was monitored by the bioluminescence of tumor cells using a live animal imaging system (PerkinElmer, IVIS Spectrum). At the end of the experiment, mice were euthanized and injected intratracheally with India ink. Then, the stained lungs were fixed in Fekete’s solution (1 mL formalin, 0.5 mL of glacial acetic acid, 10 mL of 70% ethanol) for 4 h. Subsequently, the tumor lesions appeared white, and the normal lung tissue remained black. Tumor metastatic sites in the lungs were counted carefully.

### Mouse single-cell RNA-seq data analysis

To study the difference in the tumor microenvironment, 5 × 10^5^ RM1 cells or hybrid cells were subcutaneously injected into mice, and the tumors were harvested when their volume reached ~ 200 mm^3^. The tumor specimens were cut into pieces and digested with collagenase D (3 mg/mL; Roche) and Dispase (4 mg/mL; Sigma-Aldrich) for 1 h to prepare a cellular suspension. Then, the digested cell suspension was filtered through a 40 μm filter to remove the debris. Cells were harvested by centrifugation and resuspended in PBS, before loading onto a 10 × Genomics GemCode single-cell instrument to generate single-cell Gel Bead-In-EMLlusion. The GEM generation and barcoding, cDNA amplification, and library construction were performed using Chromium Next GEM Single Cell 3’ Reagent Kits v3.1 according to the manufacturer’s protocol. The ligation products were then sequenced an Illumina HiSeq2500 (Gene Denovo Biotechnology Co., Guangzhou, China). Cell Ranger (version 3.1.0) was used to convert raw BCL files to FASTQ files, alignment, and counts quantification. The Seurat R package (version 4.1.1) was used to convert the gene count matrices to Seurat objects and downstream analysis. Cells with 500 < UMI < 50,000 & 400 < nGene < 6500 & log_10_GenesPerUMI > 0.8 & percent.mt < 10 were selected for further analysis. The total count of each cell was normalized to 10,000. The number of principal components (PCs) was adjusted to 14 to generate cell clusters that were then exhibited using Uniform Manifold Approximation and Projection (UMAP). The doublets were identified and removed using the “DoubletFinder” method. Differential expression analysis between selected clusters was performed using the “FindMarksers” method. The cell clusters were annotated manually according to the canonical cell markers from the CellMarker website. GO, KEGG, and GSEA enrichment analyses of differentially expressed genes were performed using the “clusterProfiler” R package (v4.2.2).

### CyTOF

The bone marrow from mice with bone metastases or normal mice was flushed out and resuspended in PBS. The staining of surface epitopes was performed according to the manufacturer’s instructions (Maxpar Cell Surface Staining with Fresh Fix PN400276 A1). Cisplatin was used to label the dead cells in each sample. After washing with cell staining buffer, cells were incubated with mouse FcR blocking solution for 10 min, before incubating with cell-surface antibody as listed in Additional file 1: Table S1 at room temperature for 30 min. The cells were then washed with cell staining buffer twice and fixed in 1.6% paraformaldehyde solution. After staining, cells were stored in Fix and Perm Buffer containing 125 nM Intercalator-Ir at 4 °C overnight. Before CyTOF analysis, cells were washed twice with staining buffer and resuspended in Cell Acquisition Solution. The results of CyTOF were analyzed using Premium Cytobank software.

### Murine macrophage culture

Murine macrophages were generated from the bone marrow of 6–8-week-old EGFP C57BL/6 mice according to the previous study. The bone marrow cells were isolated from the femur and tibia of the mice, and then incubated with macrophage medium (DMEM high glucose media, 10% FBS, 1 × GlutaMAX, 1 × penicillin/streptomycin) supplemented with 50 ng/mL rmM-CSF in non-tissue-culture-treated dishes for 7 days. The harvested bone marrow-derived macrophages (BMDMs) were cocultured with RM1 cells or hybrid tumor cells in a Transwell coculture system; the tumor cells were in the upper chamber, and the macrophages were in the lower chamber. After a 3-day culture, the total mRNA of the macrophages in different groups was extracted using an RNA isolation kit (Vazyme, RC112-01) according to the manufacturer’s instructions. Quantitative RT-PCR was performed to identify the expression level of M2-like macrophage marker genes in macrophages from different groups according to the manufacturer’s instructions.

### Cytokine array

For cytokine array analysis, the RM1 cells and hybrid tumor cells were cultured in DMEM high glucose medium supplemented with 2% FBS and 1 × penicillin/streptomycin for 3 days. Then, the supernatant of the RM1 and hybrid cells was harvested to perform cytokine array analysis according to the manufacturer’s instructions. In brief, 100 μL sample diluent was added to each well and incubated at room temperature for 30 min to block the slides. After removing the buffer, 100 μL of sample was added to each well and incubated at room temperature for 2 h. Then the samples were decanted, and the wells were washed with wash buffer and incubated with biotinylated antibody cocktail for 2 h followed by incubation with Cy3 equivalent dye labeled-streptavidin for 1 h. These signals were visualized using a laser scanner. The data were extracted using GenePix and further analyzed using RayBio analysis tools.

### Cell viability assay

For the cell viability assay, RM1 and hybrid cells (2000 cells/well) were seeded into 96-well plates and cultured at a 37 °C incubator for 24 h. For ferroptosis inducers, cells were treated with elastin (Selleck, 0.1 μM, 0.25 μM, 0.5 μM, 1 μM, 2.5 μM, 5 μM, 10 μM, 25 μM, 50 μM), RSL3 (MCE, 0.05 μM, 0.1 μM, 0.25 μM, 0.5 μM, 1 μM, 2.5 μM, 5 μM, 10 μM), and FIN56 (Selleck, 0.1 μM, 0.25 μM, 0.5 μM, 1 μM, 2.5 μM, 5 μM, 10 μM) for 24 h, and the matched volume of DMSO was used as a control. For the chemotherapy drug, cells were treated with docetaxel (MCE, 0.5 nM, 1 nM, 2.5 nM, 5 nM, 10 nM, 20 nM) for 48 h, with a matched volume of DMSO used as a control. For radiation sensitivity, the cells were irradiated with 6 Gy X-ray using an RS 2000 PRO 225 X-ray irradiator (Rad Source) with a single dose at 2 Gy/min, and cell viability was assessed at 0 h, 24 h, 48 h, and 72 h after irradiation. The cell viability was assayed with a Cell Count Assay Kit (Yeasen, 40203ES60) according to the manufacturer’s instructions and measured at wavelength 450 nm with a microplate reader (BioTek Synergy HTX) [23]. The cell viability was calculated and compared with the control for both cell lines.

### Clonogenic assay

RM1 cells or tumor hybrid cells were seeded into 6-well plates at densities of 200 cells/well, 400 cells/well, 1000 cells/well, and 2000 cells/well and cultured in a 37 °C incubator for 24 h. Then, the cells were irradiated with 0 Gy, 2 Gy, 4 Gy, and 6 Gy X-ray using an RS 2000 PRO 225 X-ray irradiator (Rad Source) with a single dose at 2 Gy/min according to the seeding density. After incubation for 1 week, the cells were stained with 0.5% crystal violet, and colonies with more than 50 cells were counted. The survival fraction was calculated and normalized to that of the unirradiated group. The survival curve was constructed using the multitarget single-hit model (*Y* = 1 − (1 − exp(− k*x))^*N*^) by GraphPad Prism 9.0.

### Therapy experiment

RM1 (5 × 10^5^) and hybrid tumor cells (5 × 10^5^) were suspended in 100 µL PBS. Tumor cells were injected subcutaneously into the flank of 6- to 8-week-old C57BL/6 male mice. For the bone metastasis model, the mice were injected with 1 × 10^5^ tumor cells through the caudal artery. When the tumor volume reached ~ 100 mm^3^ or there was significant bioluminescence in the bone area, the mice were randomly divided into different treatment groups, including erastin (20 mg/kg, dissolved in 5% DMSO + 40% PEG300 + 5% Tween 80 + 50% ddH_2_O, i.p., daily), RSL3 (100 mg/kg, dissolved in 10% DMSO + 40% PEG300 + 5% Tween-80 + 45% saline, i.p., biweekly), and docetaxel (12.5 mg/kg, dissolved in 10% DMSO + 40% PEG300 + 5% Tween-80 + 45% saline, i.p., biweekly), with the matched volume of vehicle solution used as the negative control. For radiotherapy, the mice were anesthetized and received local ionizing radiation on tumors at 8 Gy using an RS 2000 PRO 225 X-ray irradiator (Rad Source) with a single dose at 2 Gy/min. Tumor volume and body weight were measured every 3 days, and the volume was calculated as follows: length × width^2^/2. Mice were euthanized when the tumor volume exceeded 2000 mm^3^.

### Statistics

GraphPad Prism 9.0 and R software (4.1.0) were used for all statistical analyses. All data are presented as the mean ± SEM with no special statement. Two-tailed unpaired Student’s t-test was used to compare the difference between the two groups. Log-rank test was used to compare the survival curves. One-way ANOVA and Tukey’s posttest were used for multiple comparisons. Chi-squared (and Fisher’s exact) test was used to compare the frequencies in different groups. *P* < 0.05 was considered to indicate a statistically significant difference.

## Results

### Identification of myeloid-like disseminated cancer cells in PCa bone metastases

**Fig. 1:**
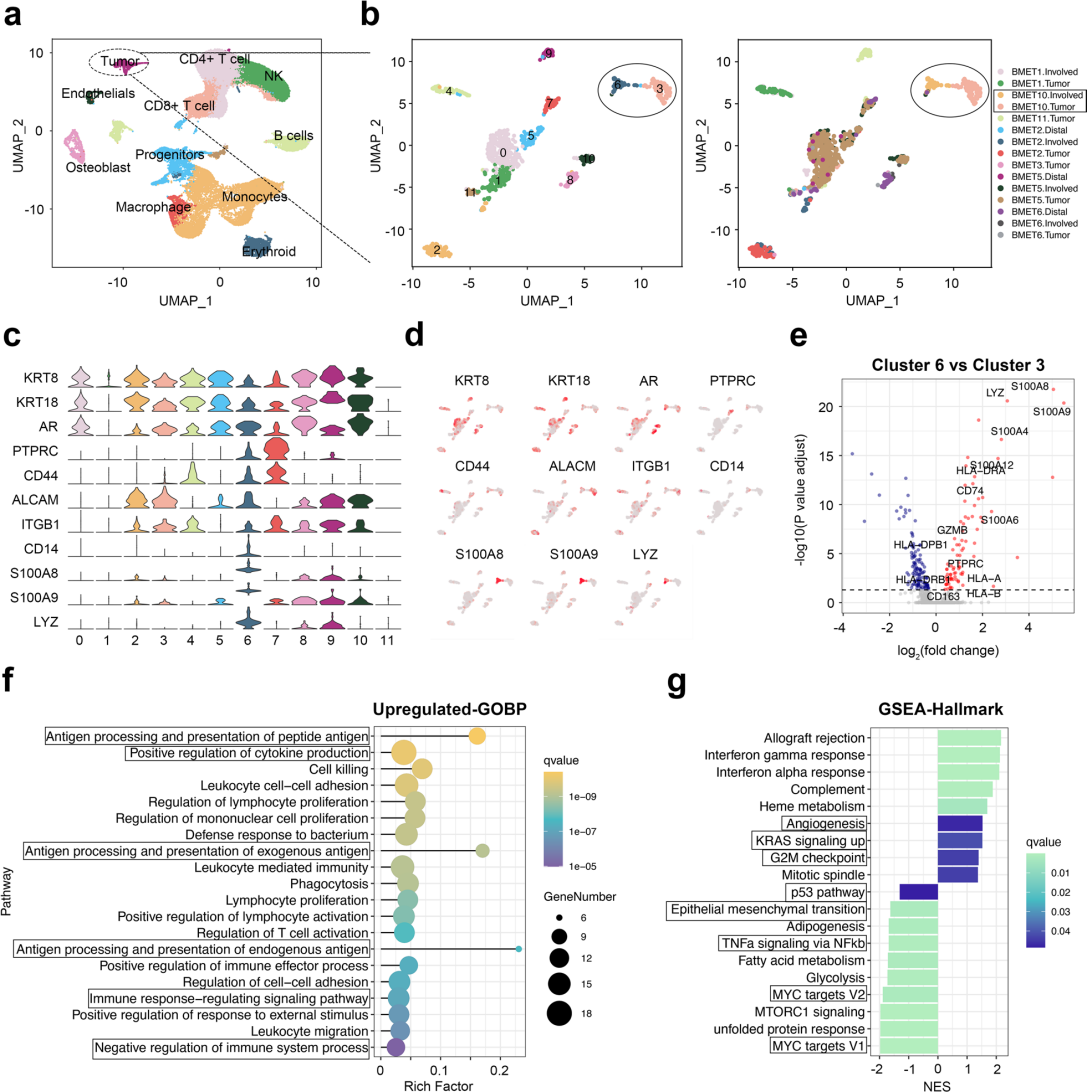
Identification of myeloid-like disseminated cancer cells in the bone marrow of patients with PCa bone metastasis. **a** UMAP plots showing the populations of 55,276 cells from the three combined scRNA-seq (tumor in bone, involved marrow, and distal marrow) of patients with PCa, colored by cell annotation. **b** UMAP plots showing the transcription heterogeneity of tumor cells colored by cell cluster (left) and sample type (right). **d** Dot plots showing the average expression level of known markers in tumor cells from different types of samples. **c** UMAP plots showing the expression level of selected genes in tumor cells. **e** Volcano plots showing the differentially expressed genes between myeloid-like tumor cells and normal tumor cells. **f** Dot plot showing GO enrichment analysis of up-regulated genes in myeloid-like tumor cells. **g** Bar plot showing the result of GSEA of hallmark pathways in cluster 6 (myeloid-like tumor cells) compared with cluster 3

To understand the status of disseminated PCa cells in bone marrow, we first analyzed the transcriptome of DTCs from a single cell RNA-sequencing data of PCa bone metastases (GSE143791) [20]. After quality control, we visualized the cells using UMAP and annotated them according to the previous results (Fig. 1a), before selecting and re-clustering the tumor cells for further analysis. According to the results of UMAP, the disseminated PCa cells can be divided into 12 subclusters, and tumor cells from different patients showed significantly different expression patterns (Fig. 1b). We analyzed the genes expressed differently in different clusters of tumor cells and found that S100A8, S100A9, and LYZ (the markers of myeloid cells) were highly expressed in cluster 6 (Fig. 1c, d, Additional file 2: Fig. S1a). To eliminate the influence of individual differences on analysis results, we selected cluster 3, which was from the same patients as cluster 6, to perform further transcriptome comparison. As a result, we found that CD163, CD74, S100A4, S100A12, HLA-DRA, and HLA-DRB1, which are usually highly expressed in myeloid cell, were also highly expressed in tumor cells of cluster 6 (Fig. 1e); thus, we termed this cluster myeloid-like tumor cells. We further performed gene ontology (GO) enrichment analysis of the differentially expressed genes (DEGs) in cluster 6 (Additional file 2: Fig. S1b, c) and found up-regulated genes were enriched in many immune-related biological processes, including antigen processing and presentation, positive regulation of cytokine production, regulation of the immune system, and regulation of T cell activation (Fig. 1f), indicating that these myeloid-like tumor cells can affect the immune response in the tumor microenvironment. Kyoto Encyclopedia of Genes and Genomes (KEGG) enrichment analysis showed the differentially expressed genes (DEGs) were mainly enriched in the antigen processing and presentation pathway, cell adhesion molecules, and the MAPK signaling pathway (Additional file 2: Fig. S1d). The Gene Set Enrichment Analysis (GSEA) of hallmark pathways showed that KRAS signaling pathway [24], angiogenesis [25], G2M checkpoint [26], p53 pathway [27], and epithelial mesenchymal transition (EMT) [28], all of which play important roles in tumor progression, were significantly changed in myeloid-like tumor cells (Fig. 1g). Taken together, these findings indicate that these myeloid-like disseminated tumor cells in the bone marrow are able to influence the progression of PCa bone metastasis.

### Cell fusion can generate myeloid-like tumor cells in bone metastasis

**Fig. 2:**
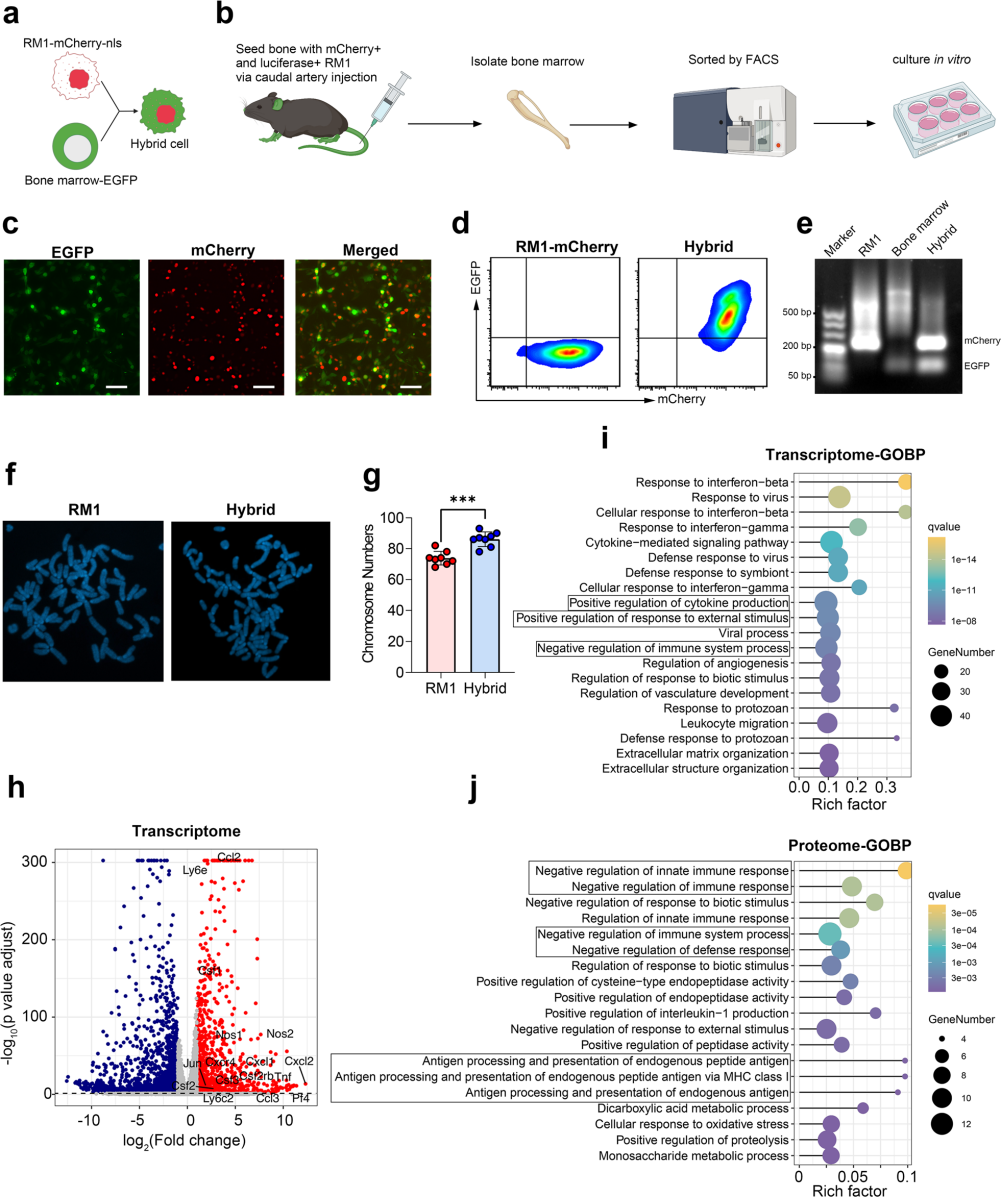
Cell fusion in the bone marrow can generate myeloid-like tumor hybrid cells. **a** Schematic illustration for identifying hybrid tumor cells. **b** Experimental schema for isolating hybrid tumor cells from the bone marrow of mice with bone metastasis. **c** Fluorescence microscope images of hybrid tumor cells.** d** Flow cytometry analysis of parental RM1 (mCherry-NLS) and hybrid tumor cells (scale bar: 50 µm). **e** Agarose gel electrophoresis of PCR products for EGFP and mCherry primers in RM1 cells, bone marrow (EGFP mouse), and tumor hybrid cells. **f** Karyotype analysis of RM1 cells and tumor hybrid cells. **g** Chromosome numbers in parental RM1 and hybrid tumor cells. **h** Volcano plot showing the differentially expressed genes between parental RM1 and hybrid tumor cells. **i**,** j** GO enrichment analysis of up-regulated genes (i) or proteins (j) in tumor hybrid cells. ***P < 0.001

Although we identified these myeloid-like tumor cells in patients with PCa, the exact impact of these cells on tumor progression still requires further experiments to prove. However, where the myeloid-like tumor cells come from and how we can obtain them remains a considerable challenge. Cell fusion is a highly regulated physiological phenomenon that takes place in development and homeostasis [29]. Spontaneous cell fusion between tumor cells or between tumor cells and other normal cells has also been widely reported in vivo, and the fused cell can acquire the phenotype from both parental cells [30–33]. Thus, we hypothesized that cell fusion between disseminated tumor cells and bone marrow cells (especially myeloid cells, because they account for the highest proportion in bone marrow) might be able to form myeloid-like disseminated tumor cells. To verify our hypothesis, we first engineered RM1 to stably express mcherry-NLS (mcherry with nuclear location sequence, to discriminate cell fusion with phagocytosis) (Fig. 2a). Then, we injected the mcherry-NLS labeled RM1 cells into EGFP C57/BL6J mice through the caudal artery to generate a bone metastasis model [22]. When bioluminescence was obvious in the bone (Additional file 2: Fig. S2a), we collected the bone marrow and sorted the double-positive cells (mCherry^+^ and EGFP^+^) by flow cytometry (Fig. 2b, Additional file 2: Fig. S2b). Following the proliferation of hybrid cells in vitro, we identified the hybrid cell by fluorescence microscopy and flow cytometry. As shown in Fig. 2c, d, the nuclear mCherry and EGFP were coexpressed in sorted cells. To further identify the integration of the genome between RM1 and bone marrow cells, we extracted genomic DNA from RM1-mCherry, bone marrow cells (EGFP mouse), and hybrid tumor cells, and performed PCR amplification of EGFP and mCherry in all of them. As shown in Fig. 2e, agarose gel electrophoresis of the PCR products showed hybrid tumor cells had two brands from EGFP and mCherry, while the RM1 and bone marrow cells had only one brand from mCherry and EGFP respectively, which indicated that hybrid cells contained genetic materials from both RM1 cells and bone marrow cells. Furthermore, karyotype analysis showed that the number of chromosomes in hybrid cells was significantly higher than that of parental RM1 (Fig. 2f, g).

To compare the difference between hybrid cells and parental RM1, we further analyzed the transcriptome and Proteome in these two types of cells. In the transcriptome, we identified 2822 DEGs in hybrid tumor cells (|Log2(fold change)|> 1, FDR < 0.05), including 1093 up-regulated genes and 1729 down-regulated genes (Fig. 2h). We found that many myeloid cell markers were highly expressed in hybrid tumor cells, including Ly6e, Ly6c2, Ccl2, Nos2, Cxcl1, and Cxcl2 (Fig. 2h). Proteome analysis showed 370 significantly different proteins in hybrid tumor cells (|Log2(fold change)|> 1, FDR < 0.05), including 152 up-regulated proteins and 217 down-regulated proteins (Additional file 2: Fig. S2c). PaGenBase database cell characteristic enrichment analysis showed that the up-regulated genes (transcriptome) in hybrid tumor cells were mainly enriched in macrophage, M1 macrophage, and mast cells, indicating that hybrid tumor cells had some characteristics of myeloid cells (Additional file 2: Fig. S2d). GO enrichment of up-regulated genes or up-regulated proteins in hybrid tumor cells revealed that a number of immune regulation pathways were significantly enriched, including response to interferon-beta, negative regulation of the immune system process, positive regulation of response to an external stimulus, cytokine-mediated signaling pathway, and antigen processing and presentation (Fig. 2i, j), which were similar to the results of myeloid-like disseminated tumor cells found in humans (Fig. 1f). Based on these results, we conclude that cell fusion between tumor cells and bone marrow cells can generate myeloid-like disseminated tumor cells in vivo.

### Multi-omics shows the difference between parental RM1 cells and tumor hybrid cells

**Fig. 3:**
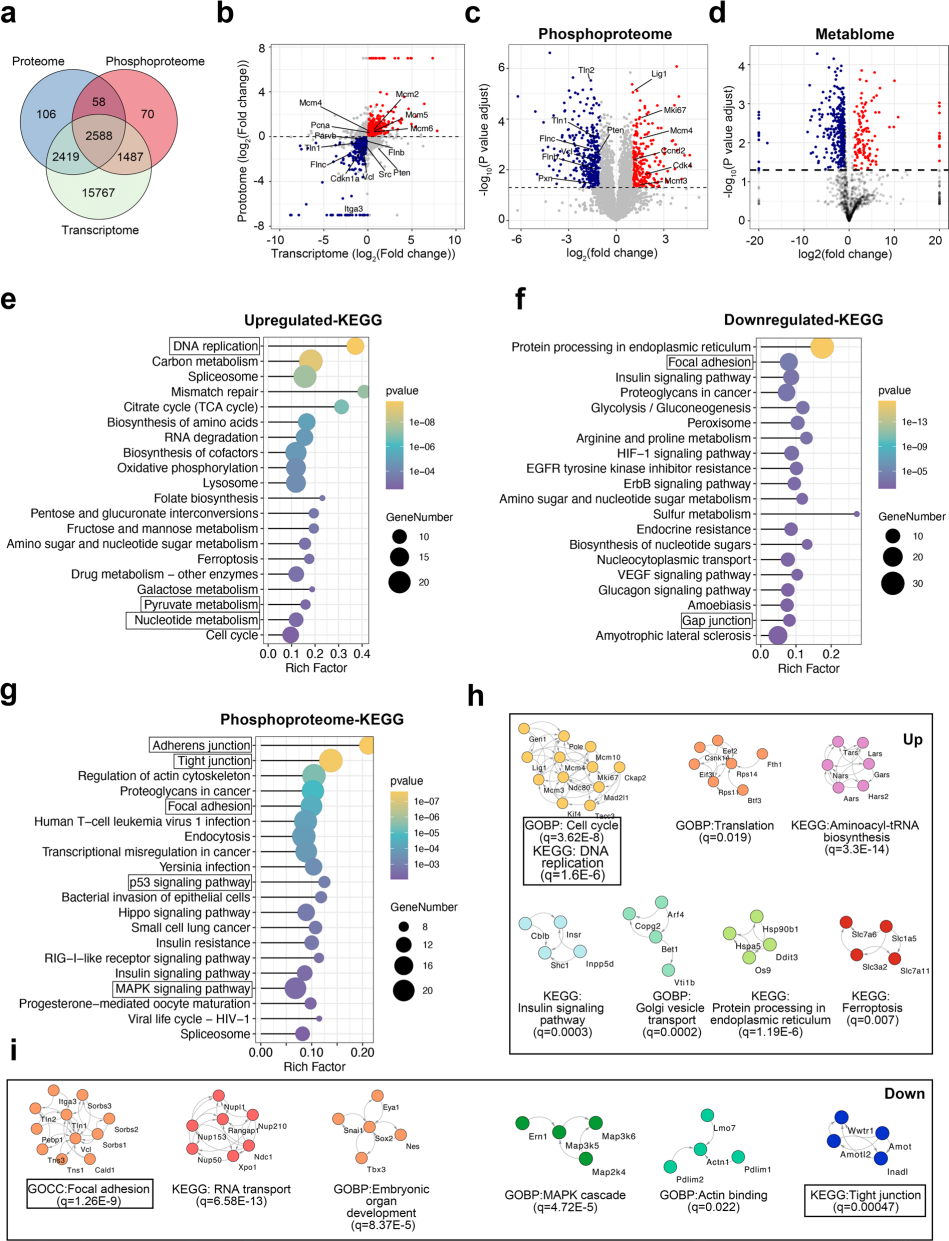
Multi-omics analysis reveals the difference between parental RM1 and hybrid cells. **a** Venn diagram showing the overlap of detected genes, proteins, and phosphoproteins. **b** Volcano plot showing the common genes in the transcriptome and proteome. **c** Volcano plot showing the proteins with different phosphorylation status between parental RM1 and hybrid tumor cells. **d** Volcano plot showing the different abundance of metabolites between RM1 cells and hybrid tumor cells. **e**, **f** KEGG enrichment analysis of the commonly up-regulated genes (**e**) and down-regulated genes (**f**) in both the transcriptome and proteome. **g** KEGG enrichment analysis of the proteins with different phosphorylation statuses between parental RM1 and hybrid tumor cells. **h**, **i** Functional STRING network of the proteins with significantly higher phosphorylation status (**h**) or lower phosphorylation status (**i**) in hybrid cells

To analyze the difference in pathways between hybrid and RM1 cells, we performed KEGG pathway enrichment analysis on the differentially expressed genes or proteins. In the transcriptome, the PI3K-AKT signaling pathway, MPAK signaling pathway, focal adhesion, and cell adhesion molecules were significantly enriched (Additional file 2: Fig. S3a). In the proteome, nucleotide metabolism, pyrimidine metabolism, and the p53 signaling pathway were significantly enriched (Additional file 2: Fig. S3b). We next combined the proteome and transcriptome data by gene names and found 5007 common genes in both omics (Fig. 3a). We found 583 entities (Log_2_(FC_protein_) > 0, Log_2_(FC_mRNA_) > 0, FDR < 0.05) were consistently higher in hybrid cells and 464 entities (Log_2_(FC_protein_) < 0, Log_2_(FC_mRNA_) < 0, FDR < 0.05) were consistently higher in RM1 cells (Fig. 3b). KEGG enrichment analysis showed the commonly up-regulated genes in hybrid cells were mainly enriched in the DNA replication, cell cycle, carbon metabolism, and nucleotide metabolism (Fig. 3e), while the commonly down-regulated genes in hybrid cells were enriched in focal adhesion, gap junction, and adherens junctions (Fig. 3f). GO BP category enrichment analysis of the transcriptome and proteome showed that the biological processes related to metabolism were highly enriched (Additional file 2: Fig. S3c–e), indicating a considerable difference in metabolism between hybrid and RM1 cells. We therefore performed metabolome analysis of hybrid tumor cells and RM1 and identified 509 differential metabolites (Fig. 3d), which were mainly enriched in amino sugar and nucleotide sugar metabolism, purine metabolism, histidine metabolism glutathione metabolism, and ferroptosis (Additional file 2: Fig. S4).

Protein phosphorylation is central to many cancer-related progresses including proliferation, metastasis, angiogenesis, and therapy resistance [34]. Thus, we performed phosphoproteome analysis of hybrid cells and RM1. As a result, we identified a total of 4203 phosphopeptides and found hybrid cells had 754 proteins with significantly different phosphorylation status, including 316 phosphorylation-up-regulated proteins and 444 phosphorylation-down-regulated proteins (Fig. 3c). KEGG pathway enrichment analysis demonstrated that the phosphorylated proteins related to adherens junction, tight junction, focal adhesion, MAPK signaling pathway, and p53 signaling pathway were mainly enriched (Fig. 3g and Additional file 2: Fig. S5). Next, we performed a functional network analysis of up-regulated proteins or down-regulated proteins separately, followed by Markov clustering (MCL), and GO/pathway enrichment in each sub-cluster (Fig. 3h, i). Among the phosphorylated proteins up-regulated in hybrid tumor cells, the major enriched terms included biological processes of cell cycle, DNA replication, aminoacyl-tRNA biosynthesis, and ferroptosis (Fig. 3h). Among the phosphorylated proteins down-regulated in hybrid tumor cells, the major enriched terms included the biological processes of focal adhesion, tight junction, and the MAPK signaling pathway (Fig. 3i).

### Myeloid-like tumor hybrid cells have an enhanced proliferative rate and metastatic capacity

**Fig. 4:**
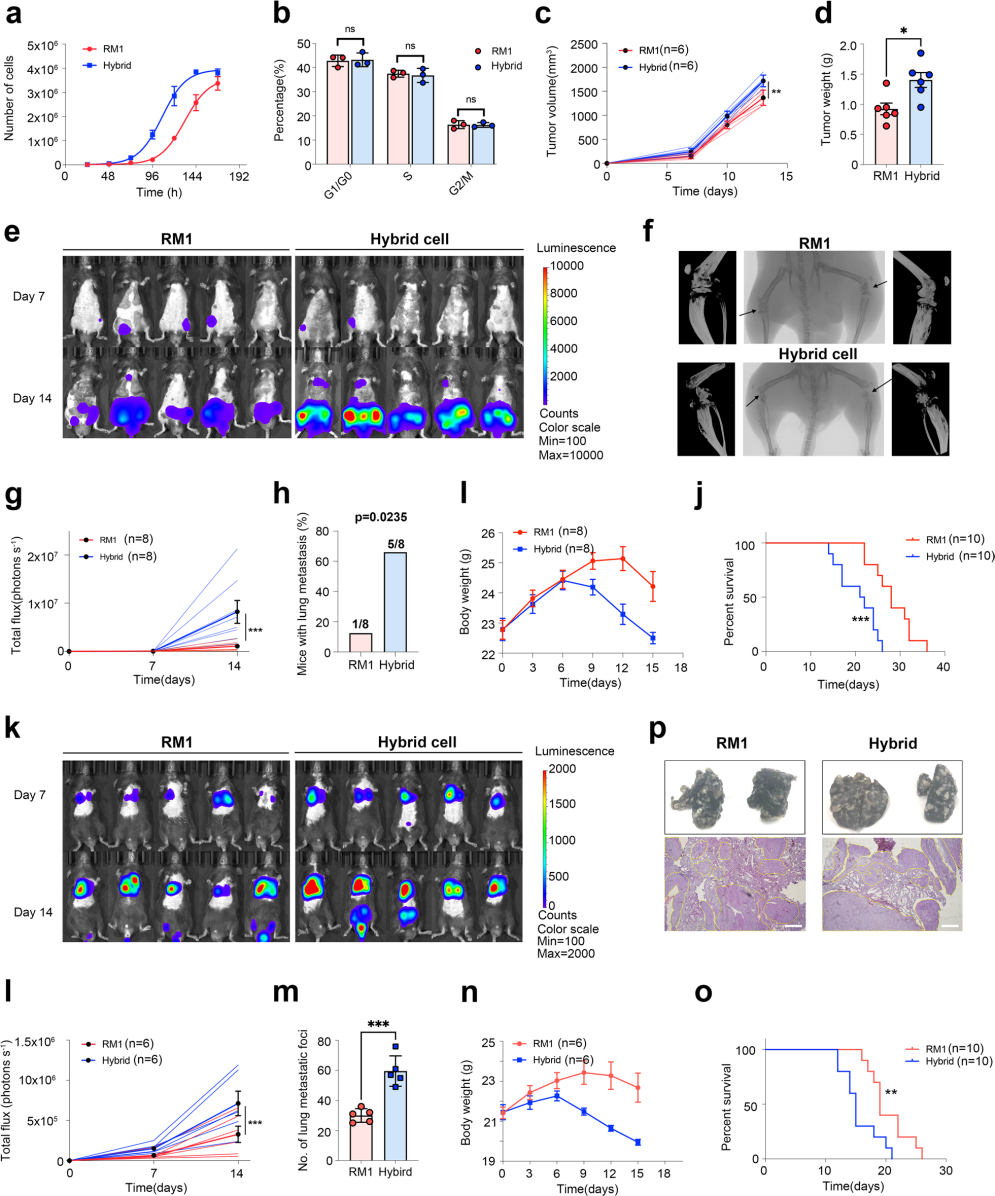
Tumor hybrid cells show a higher proliferative and metastatic capacity. **a** The growth curves of RM1 cells and hybrid cells in vitro (5000 cell/well initials, *n* = 3). **b** The proportion of RM1 cells and hybrid cells in different cell phases (*n* = 3). **c** The growth curve of RM1 tumors or hybrid tumors in vivo (5 × 10.^5^ cells per mouse, subcutaneously injected, *n* = 6). **d** Tumor weight measured at the endpoint of the experiment (*n* = 6). **e** Representative bioluminescence images of bone metastasis in mice after inoculation with RM1 or hybrid cells through the caudal artery (*n* = 8). **f** Representative micro-CT images of mice with bone metastases in the RM1 group and hybrid cell group. **g** The growth kinetics of tumor bioluminescence in mice after inoculation with RM1 or hybrid cells through the caudal artery (*n* = 8). **h** The percentage of mice with lung metastasis in the RM1 group and hybrid tumor cell group. **i** The change in body weight in mice after inoculation with RM1 or hybrid cells through the caudal artery (*n* = 8). **j** Survival curve of mice with bone metastasis in the RM1 group and hybrid cell group (*n* = 10). **k**–**o** Representative bioluminescence images (**k**), growth kinetics of bioluminescence (**l**, *n* = 6), number of lung metastatic foci (**m**, *n* = 5), body weight (**n**, *n* = 6), and survival curve (**o**, *n* = 10) of mice after inoculation with RM1 or hybrid cells through the tail vein.** p** Representative images of lungs with metastatic foci (upper, arrow indicated tumor tissue), and H&E-stained lung slices (lower) in the RM1 group and hybrid tumor cell group. *ns* Not significant, *P < 0.05; **P < 0.01; ***P < 0.001

As shown above, the genes or proteins (especially the up-regulated entities) involved in pathways related to cell proliferation, including DNA replication, and cell cycle were significantly changed among transcriptomic, proteomic, and phosphoproteomic analysis. DNA replication is a fundamental biological process in the cell cycle, which is usually dysregulated in cancer cells and can cause genomic instability [35]. GSEA also showed that the DNA replication pathway was significantly up-regulated in hybrid tumor cells (Additional file 2: Fig. S6a). The metabolism pathways related to DNA synthesis, including nucleotide metabolism, pyrimidine metabolism, were also enriched (Additional file 2: Fig. S3b). The expression of p21 (Cdkn1a) and Pten tumor suppressor genes was significantly down-regulated in hybrid cells (Fig. 3b). Furthermore, the expression of Pcna, a proliferation marker for tumor cells, was up-regulated in hybrid cells at both mRNA and protein levels (Fig. 3b). The phosphorylation status of Ki67, another proliferation marker, was significantly up-regulated in hybrid cells, while the protein level was unchanged (Fig. 3c). Taken together, these findings indicate active proliferation of hybrid cells. Thus, we tested the proliferation and cell cycle in both RM1 and hybrid cells and found a significantly higher proliferation rate of hybrid cells both in vitro and in vivo experiment, but no significant change in the proportion of cells in different cell phases (Fig. 4a–d, Additional file 2: Fig. S6b).

We observed that the KEGG pathways related to cell adhesion including focal adhesion, tight junction, and adherens junction were significantly enriched in multi-omics analysis, especially the focal adhesion pathway (Fig. 3e–g). Adhesion of cells to extracellular matrix (ECM) is central to tumor invasion and metastasis, and integrins on the cell surface can mediate adhesion to ECM and form structures called focal adhesions (FAs) [36]. Both formation and turnover of FAs are required for cell migration, and cells with more stable FAs are usually less motile and invasive, while destabilization of FAs can reduce cell adhesion to ECM and form spherical nonadherent cells [37, 38]. Talin, paxillin, vinculin, and Src form focal adhesions and were significantly down-regulated in hybrid cells (Fig. 3b); this indicated there were fewer FA structures in hybrid cells, which may be the cause of unstable adhesion of hybrid cells in vitro after several passages of culture (Additional file 2: Fig. S6c). Furthermore, we found that most MMPs, including Mmp2, Mmp3, and Mmp9, secreted by cancer cells can degrade ECM to promote cell invasion and migration [39], were up-regulated in hybrid cells (Additional file 2: Fig. S6d). Taken together, these results suggest a higher metastatic capacity in hybrid cells.

To assess the metastatic potential of hybrid cells in vivo, we first built the bone metastasis model by injection of tumor cells through the caudal artery and monitored and quantified the metastatic tumor formation using IVIS post-inoculation (Fig. 4e–j). Compared with the mice inoculated with RM1 cells, the mice inoculated with hybrid cells showed a more rapid increase in bioluminescence in bone (Fig. 4e, g), more severe bone destruction (Fig. 4f), and shorter survival time (Fig. 4j), indicating that hybrid cells had a stronger capacity for bone metastasis formation. Although caudal artery injection that delivery cancer cells through iliac artery rarely forms metastasis in other organs, we also found that some mice developed lung metastasis during the experiment (Fig. 4e), and the metastatic rate of hybrid cells was higher than that of RM1 cells (Fig. 4h), suggesting that hybrid cells might have increased capacity to disseminate to other organs or have a stronger ability to metastasize to distant sites from the bone marrow. Thus, we built the lung metastasis model by injecting tumor cells through the tail vein (Fig. 4k–p). The mice in the hybrid cell group had stronger bioluminescent signals in the lungs and increased metastasized tumor nodules on the lung surface, compared to the mice in the RM1 group (Fig. 4k–m), which was further confirmed by H&E staining (Fig. 4p). Meanwhile, the mice in the hybrid cell group had a more rapid decrease in body weight (Fig. 4n) and a shorter survival time (Fig. 4o), which indicated that hybrid cells could cause a greater severity of lung metastasis. Taken together, these results demonstrate that the hybrid cells from bone marrow had an enhanced metastasis capacity in vivo.

### Single-cell RNA-seq reveals a more immunosuppressive microenvironment in hybrid cell-derived tumors

**Fig. 5:**
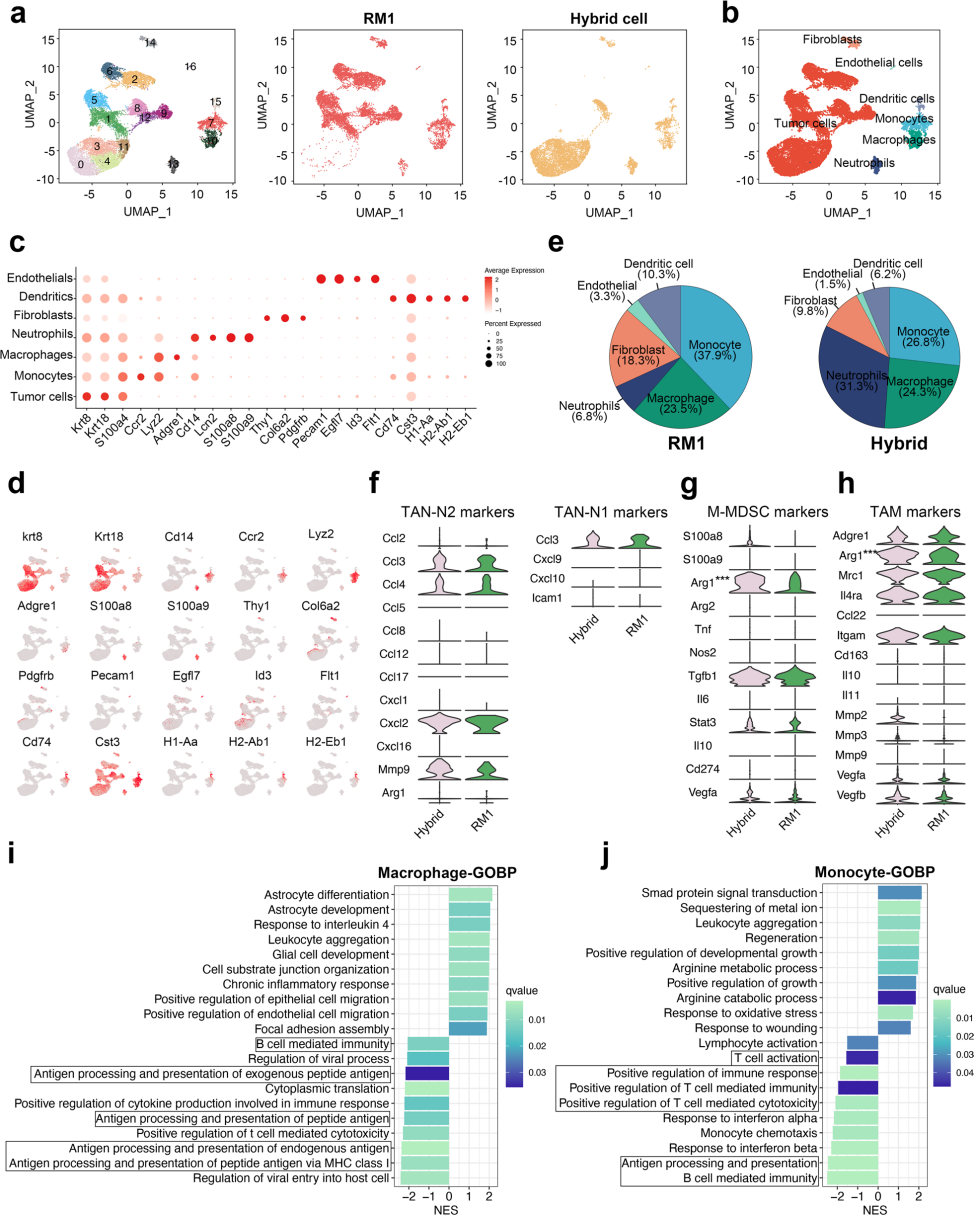
Single-cell RNA sequencing reveals the tumor microenvironment in RM1 and hybrid cell-derived tumors. **a** UMAP plots showing the 17 identified clusters of 17,602 cells from scRNA-seq of RM1-derived tumor and hybrid cell-derived tumor. The cells from RM1-derived tumor (middle) and hybrid cell-derived tumor (right) are shown. **b** UMAP plots showing the clusters colored by cell type. **c** Dot plots showing the expression level of known markers for each cell type. **d** UAMP plot showing the expression level of selected genes in all cells. **e** Pie plots showing the components of non-tumor cells colored by cell annotation. **f**–**h** Violin plot showing the expression of N1 TAN markers and N2 TAN markers in neutrophils (**f**), M-MDSC markers in monocytes (**g**), TAM markers in macrophages (**h**) from RM1-derived and hybrid cell-derived tumors. **i, j** Bar plots showing the results of GSEA of GOBP pathways in monocytes (**i**) and macrophages (**j**) from hybrid cell-derived tumors compared with those from RM1-derived tumors. ***P < 0.001

To investigate the impact of hybrid cells on the tumor microenvironment, we harvested the subcutaneous tumors formed by hybrid and RM1 cells to perform single-cell RNA sequencing. After standard data processing and quality control, we obtained transcriptional profiles from 17,602 cells and divided them 17 subclusters. UMAP was used to reduce the dimensions and visualize the cell clusters (Fig. 5a). Then, we assigned the cell clusters into seven major cell types using canonical marker genes (Fig. 5b–d and Additional file 2: Fig. S7a, b). We assessed the divergence of these two samples by viewing the same UMAP plot colored by sample types. The sample from the hybrid cell-derived tumor showed a significantly different cell distribution from that from the RM1-derived tumor (Fig. 5a). We next analyzed the cellular components of the non-tumor cells in the tumor microenvironment. Most clusters were not RM1-specific or hybrid cell-specific, indicating there was no variation in components of major cell types in non-tumor cells; however, the ratio of each cell type was significantly different, including the higher enrichment of neutrophils in hybrid-derived tumor cells (Fig. 5e). Tumor-associated neutrophils (TANs) play a dual role in tumor progression, they can be polarized to antitumor N1 TANs or protumor N2 TANs depending on the cytokine stimulation in the tumor microenvironment [40]. Thus, we analyzed the N1 and N2 markers in the neutrophils and found that the N2 markers were highly expressed in the neutrophils from both the hybrid cell group and RM1 group while the N1 markers were seldom expressed, indicating that more N2 TANs were recruited in the hybrid cell-induced tumor microenvironment (Fig. 5f). Compared with the neutrophils from RM1-derived tumors, the neutrophils in hybrid cell group showed an increased level of Il6/Jak/Stat3 signaling pathway which is usually hyperactivated in tumor infiltrating immune cells to inhibit antitumor immunity [41] (Additional file 2: Fig. S8). As macrophages and monocytes are also central to the tumor microenvironment [42, 43], we further analyzed the status of macrophages/monocytes to identify the impact of hybrid cells on macrophages/monocytes in our scRNA-seq dataset (Fig. 5g–j and Additional file 2: Fig. S7c, d). Compared with macrophages from RM1-derived tumors, GSEA of GOBP showed that macrophages from hybrid cell-derived tumors had a down-regulated antigen processing and presentation pathway, and B cell and T cell mediated immunity (Fig. 5i), which indicates that macrophages in the hybrid cell-derived tumor microenvironment have a weaker ability to activate the immune response. The GSEA of GOBP in monocytes from hybrid cell-derived tumor showed a similar result (Fig. 5j). We then analyzed some marker genes of M-MDSCs and TAMs in monocytes and macrophages, and found that Arg1 highly expressed in both monocytes and macrophages from hybrid-derived tumors (Fig. 5g, h), which can inhibit the function of T cells by depleting local L-arginine [44].

### Tumor hybrid cells can induce more M2-like macrophages in bone marrow

**Fig. 6:**
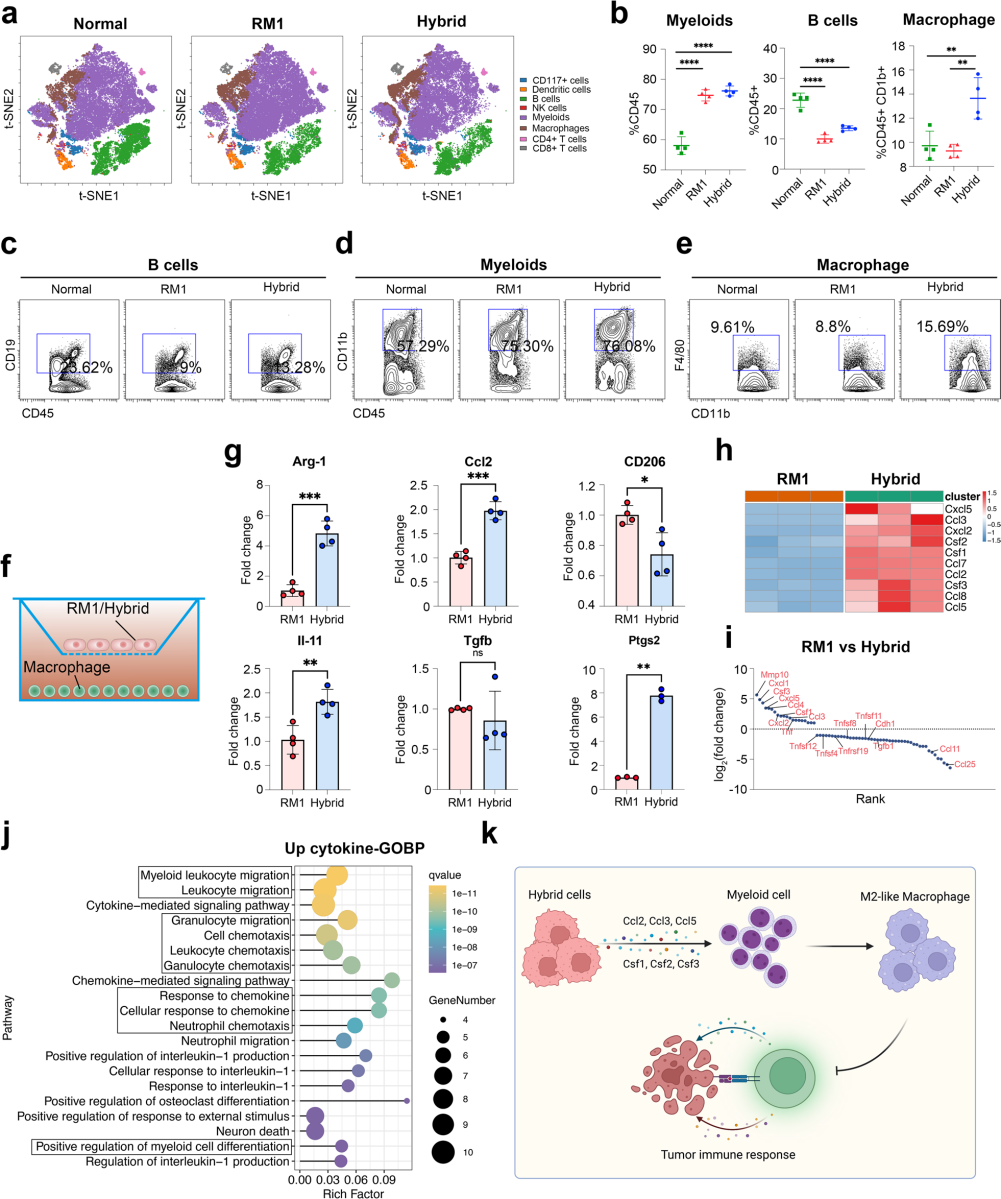
Tumor-associated macrophages show higher immunosuppressive capacity in the hybrid cell-induced microenvironment. **a** Representative tSNE plots of bone marrow from normal mice (left), mice with RM1-derived bone metastasis (middle), and mice with hybrid cell-derived bone metastasis (right). **b** The percentage of myeloid cells (left) and B cells (middle) in CD45^+^ cells, the percentage of macrophages (right) in CD45^+^ CD11b.^+^ cells (*n* = 4). **c**–**e** Representative CyTOF dot plots showing the percentage of B cells (**c**), myeloid cells (**d**), and macrophages (**e**) in different groups. **f** Schematic illustration of macrophages cocultured with RM1 or hybrid cells.** g** qRT–PCR for Arg-1, Ccl2, Cd206, Il-11, Tgfb, and Ptgs2 expression in macrophages cocultured with RM1 or hybrid cells (*n* = 4). **h** Heatmap showing the expression of chemokines and cytokines related to myeloid cell recruitment and differentiation in RM1 cells and hybrid cells. **i** Cytokine array analysis of cell culture supernatant from RM1 cells and hybrid cells. **j** GO enrichment analysis of up-regulated cytokines (Log_2_(fold change) > 1) in hybrid cells, based on the result of cytokine array. **k** High expression of Ccl2, Ccl3, Ccl5, Csf1, Csf2, and Csf3 in hybrid cells can recruit myeloid cells to the tumor, and then induce them to polarize into N2-like neutrophils or M2-like macrophages to inhibit the immune response. ns: not significant, *P < 0.05; **P < 0.01; ***P < 0.001; ****P < 0.0001

The bone marrow microenvironment is important for bone metastasis formation [5], thus we further investigated the impact of hybrid cells on bone marrow cells. We injected the tumor cells into mice through the caudal artery and harvested the bone marrow cells to perform CyTOF when there was significant bone metastasis, while the bone marrow from normal mice was used as control. The viSNE analysis showed that there were at least eight populations according to our gating strategy among all three groups (Fig. 6a and Additional file 2: Fig. S9). Then, we compared the proportion of each population among these three groups and found a consistent decrease in B cells (CD45^+^ CD19^+^) and increase in myeloid cells (CD45^+^ CD11b^+^), in mice with bone metastasis (Fig. 6b–e). Compared with mice with RM1-derived bone metastasis, the mice with hybrid cell-derived metastasis showed a significantly increased frequency of macrophages (Fig. 6b). To identify the impact of hybrid cells on macrophage differentiation, we co-cultured RM1 and hybrid cells with bone marrow-derived macrophage for 3 days and then tested the expression of M2 macrophage marker genes (Fig. 6f). The results showed that the expression level of Arg1 was significantly higher in the macrophages co-cultured with hybrid cells, which were identical to the results of scRNA-seq shown above (Fig. 6g). PGE2 can inhibit anti-tumor immunity in tumor microenvironment [45], PTGS2, as the key enzyme in PGE2 biosynthesis, was also highly expressed in macrophages co-cultured with hybrid cells (Fig. 6g).

To investigate the mechanism by which hybrid cells induce M2-like macrophages, we analyzed the secretome transcripts of both RM1 and hybrid cells. Among the nearly 300 differentially expressed secreted chemokines and cytokines (Additional file 2: Table S1), we found that Cxcl2, Cxcl5, Ccl2, Ccl3, Ccl5, Ccl8, Csf1, Csf2, and Csf3 were among the top up-regulated secreted factors (Fig. 6h), it has been widely reported that Ccl2, Ccl5 and Csf1 can promote the recruitment of macrophages in tumor sites, Csf1 also can induce tumor-associated macrophages to polarized into M2 phenotype [46]. Furthermore, Cxcl5 and Cxcl2 can promote the recruitment of neutrophils, Ccl2, Ccl7 and Ccl8 can promote the recruitment of monocytes, and Ccl5 can promote the recruitment of granulocytes [46]. The GO enrichment analysis of upregulated chemokines and cytokines showed that these factors were mainly included in myeloid leukocyte recruitment, like neutrophils chemotaxis and granulocyte chemotaxis (Additional file 2: Fig. S10). Cytokine array analysis of supernatant from RM1 and hybrid cells showed a similar result (Fig. 6i), the highly secreted cytokines in hybrid cells were mainly enriched in the pathway related myeloid cell migration and chemotaxis (Fig. 6j). Taken together, the cytokines secreted by hybrid cells can recruit the myeloid cells to tumors and induce them to polarize to N2-like neutrophils or M2-like phenotype to inhibit the immune response (Fig. 6k). Cxcl2, Ccl2, Ccl3, and Ccl8 can act as chemoattractants for myeloid cell recruitment in tumors [46], while Csf1, Csf2, and Csf3 are central to controlling the recruitment, proliferation, and differentiation of immunosuppressive myeloid cells [46], including macrophages and neutrophils in the tumor.

### Hybrid cells show an enhanced EMT phenotype with higher tumorigenic potential

**Fig. 7:**
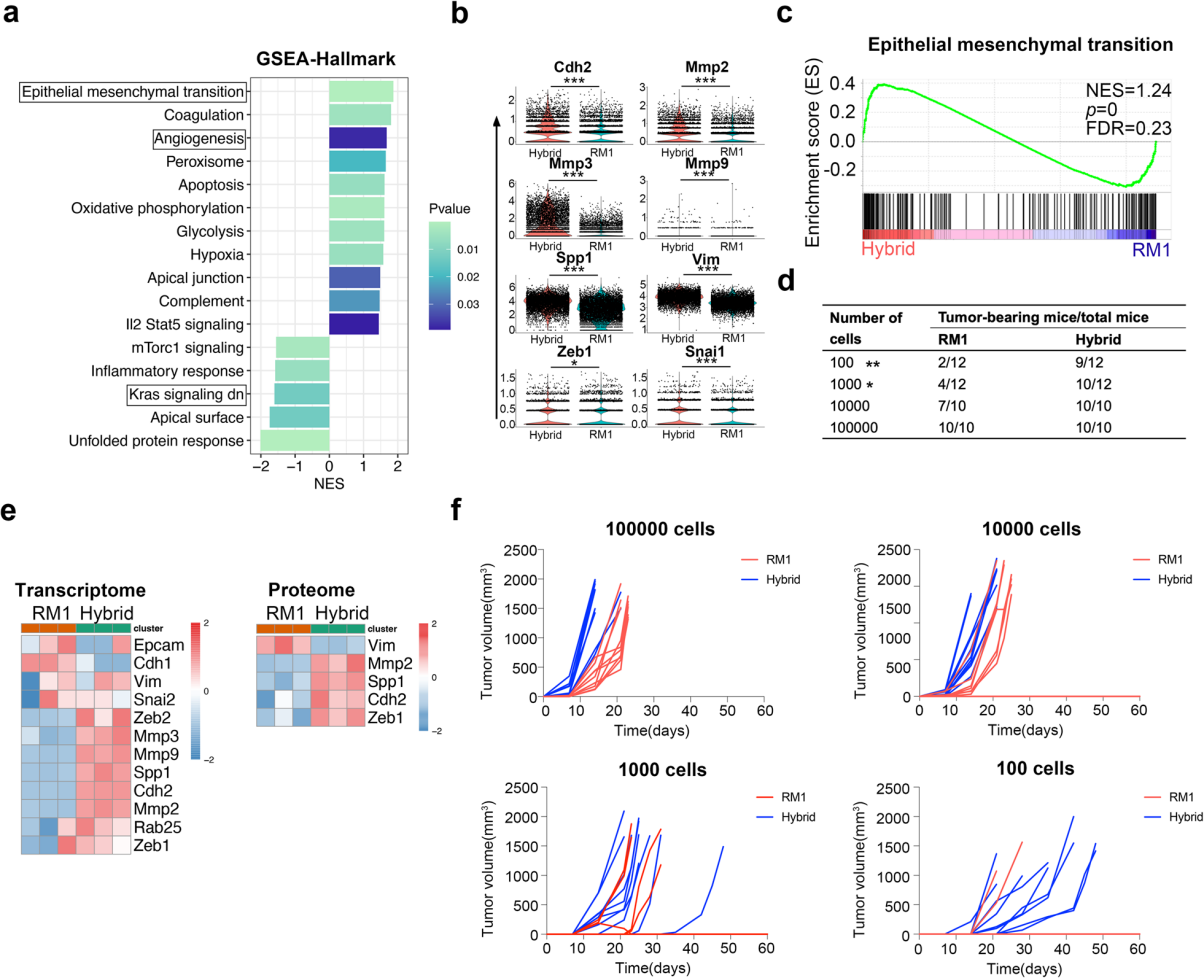
Tumor hybrid cells show an enhanced EMT pathway with higher tumorigenic potential in vivo. **a** Bar plot showing the result of GSEA of hallmark pathways in tumor cells in hybrid cell-derived tumors. **b** Violin plots showing the expression of EMT marker genes in tumor cells from RM1-derived tumors and hybrid cell-derived tumors. **c** GSEA of EMT pathways in hybrid tumor cells compared to RM1 cells.** d** Tumor-initiating capacity of parental RM1 cells and hybrid tumor cells. **e** Heatmap showing the differentially expressed genes or proteins in the EMT pathway between RM1 cells and hybrid tumor cells. **f** Tumor growth curves and tumor formation rate of mice injected subcutaneously with different numbers of tumor cells (*n* = 10). *P < 0.05, **P < 0.01, ***P < 0.001

We further compared the hallmark pathways in tumor cells from RM1-derived tumor and hybrid cell-derived tumor based on scRNAseq. The pathway analysis revealed that the epithelial mesenchymal transition (EMT) pathway, which was associated with many processes in tumor including tumor initiation and metastasis [47], was most significantly up-regulated in hybrid cell-derived tumor cells in vivo and in vitro (Fig. 7a–c), and the marker genes involved in EMT, including Cdh2 (N-Cadherin), Mmp2, Mmp3, Vim, and Spp1, were highly expressed in hybrid tumor cells (Fig. 7e), which suggested hybrid cells might have a stem-like phenotype with high metastatic and tumorigenic potential in vivo. To assess the tumorigenic potential of hybrid cells in vivo, we injected 100,000, 10,000, 1000, or 100 cells of hybrid cells/RM1 cells subcutaneously into EGFP C57/BL6J mice, respectively, and tumors were examined for 2 months post-inoculation. There was no significant difference in tumor formation rate between hybrid and RM1 cells in mice injected with 100,000 or 10,000 cells (Fig. 7d). The tumor formation rate of hybrid cells was significantly higher than that of RM1 cells in mice injected with 1000 or 100 cells (Fig. 7d, f), indicating that hybrid cells had more robust tumorigenicity than parental RM1 cells in vivo.

### Myeloid-like tumor hybrid cells are resistant to docetaxel and ferroptosis inducers, but sensitive to radiotherapy

Multidrug resistance is a major problem in patients with bone metastasis, which further affects the treatment efficiency of chemotherapy [48]. It has also been reported that EMT is related to drug resistance [49]. Docetaxel is the main chemotherapy drug for patients with metastatic castration-resistant PCa [50]; thus, we next compared the docetaxel sensitivity of hybrid cells and parental RM1 cells in vitro. The IC_50_ values of docetaxel for hybrid and RM1 cells were determined by cytotoxicity assay based on the CCK8 cell proliferation assay following drug treatment for 48 h. As shown in Fig. S11a, the dose–response curve showed that hybrid cells (IC_50_: 5.94 nM) were less sensitive to docetaxel than parental RM1 cells (IC_50_: 3.31 nM). To further test the docetaxel sensitivity of hybrid cells in vivo, the mice were injected with the tumor cells subcutaneously and then treated with docetaxel (12.5 mg/kg) or vehicle twice a week when the tumor volume reached ~ 100 mm^3^ (Additional file 2: Fig. S11b). In accordance with the results in vitro, treatment of docetaxel led to significant inhibition of tumor growth in the RM1 group, but not in the hybrid cell group based on the tumor growth curve and tumor weight (Additional file 2: Fig. S11c–e). These results indicate that myeloid-like tumor hybrid cells are less sensitive to docetaxel in vivo, which suggests that cell fusion is one of the reasons causing drug resistance in bone metastasis.

**Fig. 8:**
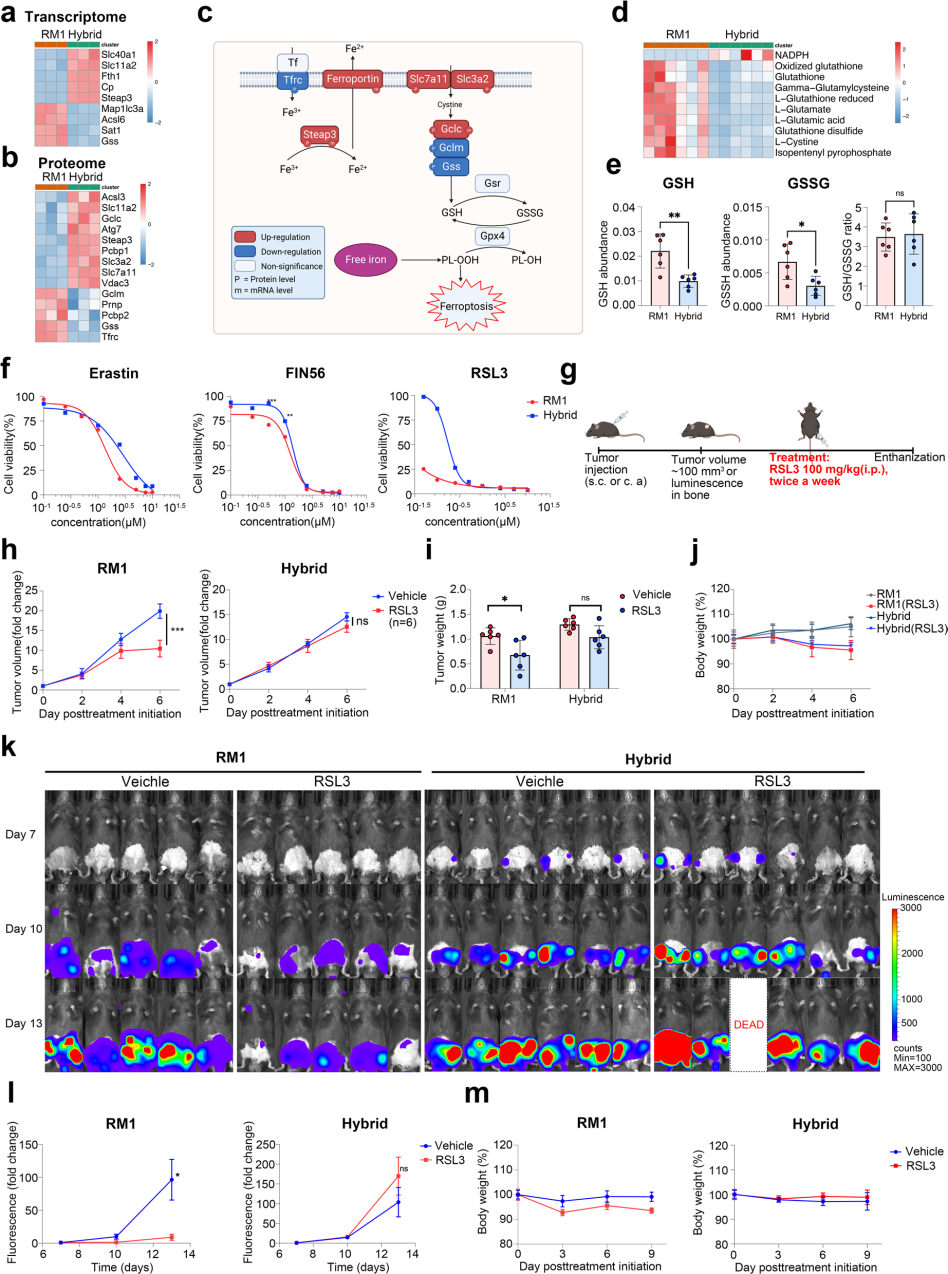
Tumor hybrid cells are resistant to ferroptosis in vitro and in vivo. **a**, **b** Heatmap showing the differentially expressed genes (**a**) or proteins (**b**) in the ferroptosis pathway between RM1 cells and hybrid tumor cells. **c** Diagram showing the changes in key genes involved in ferroptosis. **d** Heatmap showing the differential metabolites related to ferroptosis between RM1 cells and hybrid cells. **e** The level of GSH, GSSH, and GSH/GSSH in RM1 cells and hybrid cells. **f** Viability of RM1 cells and hybrid cells after erastin, FIN56, or RSL3 treatment. **g** Schematic illustration of the experimental design using RSL3 treatment. **h** The growth curve of RM1 tumor or hybrid tumors after treatment with RSL3 (*n* = 6). **i** Tumor weight measured at the endpoint of the experiment after treatment with erastin (*n* = 6). **j** The growth curve of body weight in mice treated with RSL3 or vehicle (*n* = 6). **k**, **l** Representative bioluminescence images (**k**) and growth kinetics (**l**) of bioluminescence in mice after inoculation with RM1 (*n* = 5) or hybrid cells (*n* = 6) through the caudal artery. **m** The growth curve of body weight in mice treated with RSL3 or vehicle (*n* = 5 or 6). ns: not significant, *P < 0.05; **P < 0.01; ***P < 0.001

Ferroptosis is a newly identified iron-dependent programmed cell death mechanism characterized by increased lipid peroxidation [51]. It has been reported that ferroptosis is involved in chemotherapy, radiotherapy, and immunotherapy; therefore, ferroptosis activation is a potential method to overcome drug resistance [52]. Thus far, ferroptosis inducers have shown promising antitumor efficiency in preclinical trials of multiple cancers, including PCa [53]. We found that many differentially expressed genes and proteins were enriched in ferroptosis and glutathione metabolism in KEGG enrichment analysis (Fig. 8a, b). The ferroptosis suppressors Slc7a11, Slc3a2, Slc40a1, and Gclc were significantly up-regulated in hybrid cells (Fig. 8c). Metabolome analysis showed a reduced level of GSH and GSSG in hybrid cells, but the GSH/GSSG ratio was not significantly different between hybrid cells and parental RM1 (Fig. 8d, e). To identify the ferroptosis sensitivity of hybrid cells, we treated the RM1 and hybrid cells with different doses of the ferroptosis inducers erastin, RSL3, and FIN56 in vitro. The dose–response curve showed that hybrid cells were less sensitive to all three ferroptosis inducers compared with the parental RM1 cells (Fig. 8f). We then further tested the ferroptosis sensitivity of the hybrid cells in vivo. The mice were injected with the tumor cells subcutaneously or through caudal artery, and were then treated with RSL3 (100 mg/kg, twice a week) or vehicle (Fig. 8g). Treatment with RSL3 led to significant inhibition of tumor growth in both subcutaneous model and bone metastasis model of the RM1 group, but not in those of the hybrid cell group, based on the growth kinetics of tumor volume and tumor bioluminescence (Fig. 8h–m). The mice treated with erastin (20 mg/kg, once daily) showed similar results (Additional file 2: Fig. S12). All of these indicate that hybrid cells are less sensitive to ferroptosis in vivo.

**Fig. 9:**
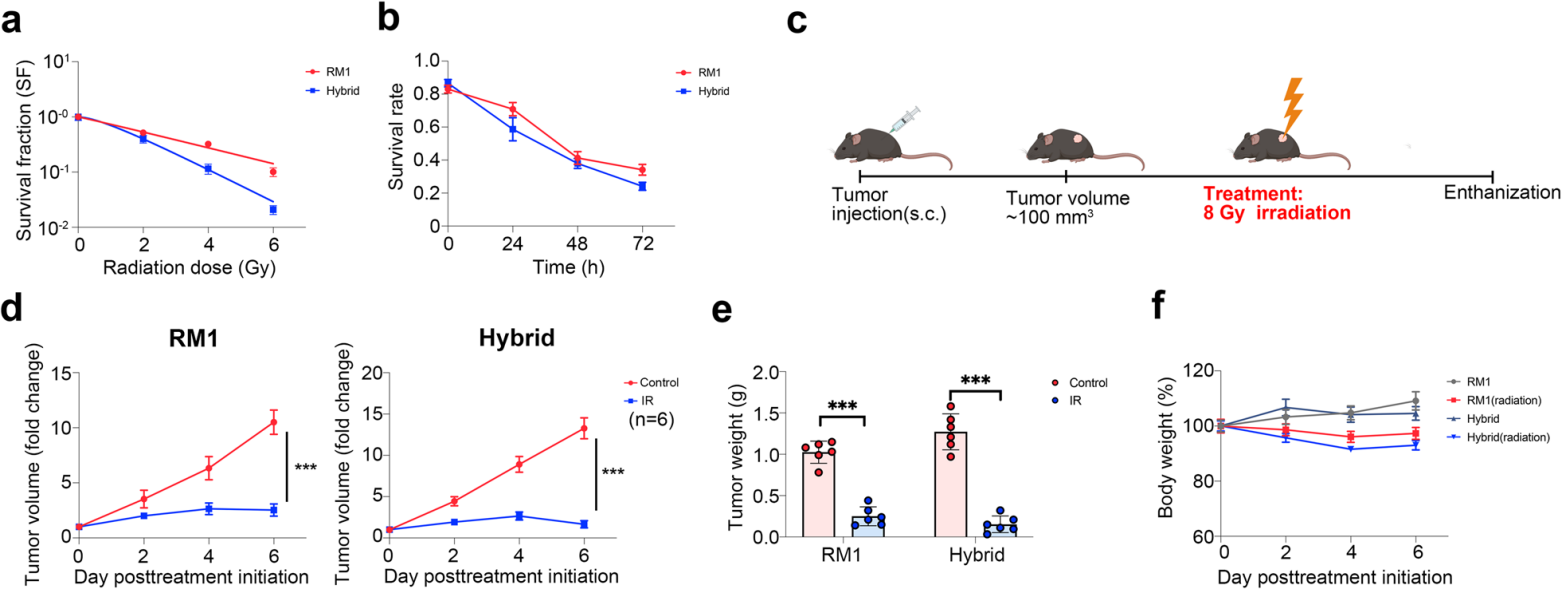
Tumor hybrid cells are sensitive to radiotherapy. **a** Clonogenic survival curves were constructed according to the survival fractions of RM1 and hybrid cells after receiving different doses of irradiation. **b** Survival rate of RM1 cells and tumor hybrid cells at different time points after receiving 6 Gy irradiation, normalized to the untreated group. **c.** Schematic illustration of the experimental design used for radiotherapy. **d** The growth curve of RM1 tumor or hybrid tumors after treatment with 8 Gy irradiation (*n* = 6). **e** Tumor weight measured at the endpoint of the experiment after treatment with radiotherapy (*n* = 6). **f** The growth curve of body weights in mice receiving radiotherapy and not (*n* = 6). ***P < 0.001

Radiotherapy is an important palliative treatment for patients with PCa with bone metastasis [54]. Thus, we further investigated the radiotherapy sensitivity of hybrid cells. Compared with parental RM1 cells, the hybrid cells showed more significant inhibition in cell proliferation and colony formation after 6 Gy X-ray treatment (Fig. 9a, b). To test the radiotherapy sensitivity of hybrid cells in vivo, the mice were injected with tumor cells subcutaneously, before treating with a single dose of 8 Gy X-ray when the tumor volume reached ~ 100 mm^3^ (Fig. 9c). Both tumors in the RM1 group and hybrid cell group were significantly inhibited (Fig. 9d–f), which indicated that radiotherapy remained an effective treatment for hybrid cells in vivo.

## Discussion

Spontaneous cell fusion between tumor cells or between tumor cells and other normal cells has been reported both in vivo and in vitro [55, 56]. The tumor hybrid cells usually obtain novel properties, while the common genotype from the parental cells is retained. The hybrid cells can cause increased proliferation and migration, immune escape, and drug resistance in tumor progression [30]. In this study, we identified a cluster of myeloid-like disseminated tumor cells in human PCa bone metastasis, and further analysis showed that these cells were significantly altered in pathways related to immune regulation and tumor progression. We found that tumor hybrid cells harvested from the bone marrow of mice with bone metastasis also showed myeloid-like phenotype based on transcriptome and proteome analyses, which indicates that cell fusion between disseminated tumor cells and bone marrow cells may be a source of the myeloid-like tumor cells. Although the mechanisms that regulate cell fusion are not yet clear, it has been reported that calcium ions are required for cell fusion [57], and hypoxia can also enhance the rate of cell fusion [58]. Bone marrow has a natural hypoxia environment with a high concentration of calcium ions, which provides a good environment for cell fusion.

Multi-omic analysis showed that the DEGs between hybrid cells and parental RM1 cells were mostly enriched in KEGG pathways related to cell proliferation and cell adhesion. The higher expression of Pcna and the higher phosphorylation of Ki67 indicated a more active proliferation status in hybrid cells. P21 can promote cell cycle arrest in the G1 phase [59]; Pten can inhibit prostate tumor cell proliferation by inhibition of PI3K/Akt pathway [60–62]; Ki67 is central to the cell cycle; and high phosphorylation of Ki67 usually occurs in mitosis cells, all of which indicated that there is an enhanced proliferation of hybrid cells. Loss of cell–cell adhesion or cell-ECM adhesion is an important process for tumor metastasis [63]. The up-regulated expression of Mmps genes and down-regulated expression of focal adhesion genes including Talin, paxillin, vinculin, and Src, in tumor hybrid cells, indicated a greater ability of hybrid cells to detach from the tumor tissue and form distant metastasis. Thus, we tested the proliferative and metastatic potential of hybrid cells. The results showed that hybrid cells had a higher proliferative rate in vitro and in vivo and had a higher metastatic capacity in both the lung metastasis and bone metastasis models. Meanwhile, we observed a high metastatic rate of hybrid cells from the bone to the lung, which means hybrid cells have a stronger ability to metastasize to distant sites from the bone marrow, and cell fusion can partly explain why the bone marrow represents a transfer station for further metastasis in PCa.

We analyzed the impact of hybrid cells on the tumor microenvironment by CyTOF and single-cell RNA sequencing. CyTOF analysis showed that the proportion of macrophages was significantly higher in bone marrow with hybrid cell-induced bone metastasis, and the macrophages in the hybrid cell-induced microenvironment showed a higher immunosuppressive capacity with higher expression of M2 marker Arg1, which can inhibit the proliferation of CD8+ T cells by depleting arginine [44, 64]. Single-cell RNA sequencing analysis showed that the infiltration of N2 TANs was significantly higher in a hybrid cell-derived tumor microenvironment. N2 TANs can promote the growth and metastasis of tumors by secreting enzymes or cytokines that can reconstruct the extracellular matrix, promote angiogenesis, and facilitate immune escape [40, 65]. The secretome analysis based on RNA-seq and cytokine array showed that the expression of chemokines that can recruit neutrophils, monocytes, and macrophages, including Ccl2, Ccl3, Cxcl1, Cxcl3, and Cxcl5, was significantly up-regulated in hybrid cells. The expression of Csf1, Csf2, and Csf3, which can induce them to differentiate into an immunosuppressive phenotype, was also up-regulated in hybrid cells. Taken together, these results indicate that hybrid cells can induce a more immunosuppressive microenvironment in vivo*,* suggesting that cell fusion can cause immune escape in vivo [66–69].

Epithelial–mesenchymal transition (EMT), in which epithelial cells acquire mesenchymal features, occurs in many physiological processes, including embryonic development, adult tissue regeneration, and tumor progression. In cancer, EMT is associated with tumor initiation, metastasis, and therapy resistance [47, 70, 71]. In our data, GSEA showed that the EMT pathway was significantly up-regulated in hybrid cells in vitro and in vivo, which could partly explain why tumor hybrid cells had a higher metastatic capacity in vivo. Hybrid cells also have a higher tumorigenic capacity in vivo and can form tumors even with only 100 cells. Based on these findings, tumor cells fused with bone marrow cells in vivo can acquire stem-like properties, which provide a better seed for bone metastasis formation and further metastasis in distant organs. Otherwise, the hybrid cells show resistance to chemotherapy, including docetaxel and ferroptosis inducers, but are sensitive to radiation. As the result of scRNAseq of human PCa bone metastasis, the myeloid-like tumor cells showed a downregulated EMT phenotype (Fig. 1g), which is inconsistent to what we found in mouse model (Fig. 7). EMT is a very complex process with intermediary or partial EMT states, called EMT heterogeneity [72]. Recent study revealed that tumor cells with hybrid E/m phenotype (epithelial-type cells with restricted mesenchymal transition) have the strongest ability to form metastases [73]. In human single cell RNAseq data, the comparison was performed between bone disseminated tumor cells, not with the primary tumor cells, which might cause the paradox result in this study. Thus, although EMT is downregulated in human myeloid-like tumor cells, they might also have stronger metastases formation ability.

## Conclusions

In summary, cell fusion between disseminated tumor cells and bone marrow cells can generate myeloid-like tumor hybrid cells with a totally different transcriptome, proteome, phosphoproteome, and metabolome. The hybrid cells can induce a more immunosuppressive microenvironment by recruiting and inducing immunosuppressive N2 TANs and M2 TAMs in vivo. Tumor hybrid cells also demonstrated a significant up-regulation in the EMT pathway, with higher tumorigenic and metastatic capacity in vivo*.* Otherwise, hybrid cells are resistant to docetaxel and ferroptosis inducers in vitro and in vivo. Based on these results, hybrid cells can promote the progression of PCa in vivo, and thus represent a potential target for PCa therapy.

## Supplementary Information

**Additional file 1: Table S1.** Differentially expressed genes of secretome.

**Additional file 2: Figure S1.** Comparison of myeloid-like disseminated cancer cells with other disseminated cancer cells. a Heatmap showing the top genes characterized in each cluster. b Heatmap showing the clustered enriched GO BP terms in the differential expressed genes in the cluster 6 versus cluster 3 differential expression analysis. c, d Dot plot showing GO enrichment analysisand KEGG enrichment analysisof differentially expressed genes between cluster 6and cluster 3. **Figure S2.** The tumor hybrid cells from bone marrow. a Representative bioluminescence image of mouse with bone metastasis after inoculation with RM1through the caudal artery. b Representative flow cytometry dot plot showing the hybrid cells in bone marrow from mice with bone metastasis. c Volcano plot showing the differentially expressed proteins between parental RM1 and hybrid tumor cells. d Enrichment analysis of up-regulated genes in PaGenBase performed by Metascape. **Figure S3.** Multi-omic analysis of RM1 cells and tumor hybrid cells. a, b Dot plot showing KEGG enrichment analysis of differentially expressed genesor proteins between tumor hybrid cells and RM1 cells. c–e Heatmap showing the clustered enriched GO BP terms in transcriptome analysis, peoteome analysis, and phosphoproteome analysis. **Figure S4.** The change of metabolism in tumor hybrid cells. a Dot plot showing the metabolic pathways in KEGG enrichment analysis of differentially expressed genes between tumor hybrid cells and RM1 cells. b Bar plot showing the result of GSEA of metabolic pathways in tumor hybrid cells compared with parental RM1 cells. c OPLS-DA scores plot of RM1 cells and tumor hybrid cells. d Validation of OPLS-DA model. e Classification of differential metabolites between tumor hybrid cells and RM1 cells. f Bar plot showing the classification of up-regulated metabolites and down-regulated metabolites in tumor hybrid cells. g Dot plot showing KEGG enrichment of differential metabolites between hybrid tumor cells and parental RM1 cells. **Figure S5.** The change in MAPK signaling pathway and p53 signaling pathway. The differentially expressed genes, proteins, or proteins with different phosphorylation statusesin MAPK signaling pathway and p53 signaling pathway. **Figure S6.** The change of DNA replication and cell adhesion in tumor hybrid cells. a GSEA of DNA replication pathways in tumor hybrid cells compared RM1 cells. b Cell cycle analysis of RM1 and tumor hybrid cells. c The morphological change of tumor hybrid cells after long term cultured in vitro, most hybrid cells became suspension growth. d The differentially expressed MMPs family genes between RM1 cells and hybrid cells. **Figure S7.** Single-cell RNA sequencing of RM1-derived tumor cells and hybrid cell-derived tumor cells. a Heatmap showing the top genes characterized in each cluster. b UMAP plots showing the clusters colored by cell type in RM1-drived tumorand hybrid cell-derived tumor. c, d Bar plots showing the results of GSEA of KEGG pathways in macrophagesand monocytesfrom hybrid cell-derived tumors compared with those from RM1-derived tumors. **Figure S8.** Bar plot showing the results of GSEA analysis of hallmark pathwayand KEGG pathwayin neutrophils from hybrid cell group compared those from RM1 group. **Figure S9.** Gating strategy for each cluster of cells in the bone marrow from normal mice, mice with RM1-derived bone metastasis, and mice with hybrid cell-derived bone metastasis. **Figure S10.** The secretome analysis of RM1 and hybrid tumor cells. a Heatmap showing the differentially expressed chemokines and cytokines between parental RM1 and hybrid tumor cells. b, c GO enrichment analysis of up-regulated chemokines and cytokinesor downregulated chemokines and cytokinesin tumor hybrid cells. **Figure S11.** Tumor hybrid cells are resistant to docetaxel in vitro and in vivo. a Viability of RM1 and hybrid cells after docetaxel treatment. b Schematic illustration of the experimental design using docetaxel treatment. c The growth curve of RM1 tumor or hybrid tumors after treatment with docetaxel. d Tumor weight measured at the endpoint of the experiment after treatment with docetaxel. e The growth curve of body weight in mice treated with docetaxel or vehicle. ns Not significant, *P < 0.05; **P < 0.01; ***P < 0.001. **Figure S12.** Tumor hybrid cells are resistant to erastin in vivo. a The growth curve of RM1 tumor or hybrid tumors after treatment with erastin. b Tumor weight measured at the endpoint of the experiment after treatment with erastin. c The growth curve of body weight in mice treated with erastin or vehicle. ns Not significant, *P < 0.05; **P < 0.01, ***P < 0.001.

## Abbreviations

ATCC: American Type Culture Collection
ANOVA: Analysis of variance
BMDMs: Bone marrow-derived macrophages
CCK8: Cell counting kit-8
CyTOF: Cytometry by time-of-flight
DEGs: Differential expressed genes
DEPs: Differential expressed proteins
DMSO: Dimethyl sulfoxide
DMEM: Dulbecco’s modifed Eagle’s medium
DTCs: Disseminated tumor cells
ECM: Extracellular matrix
EMT: Epithelial mesenchymal transition
FA: Focal adhesions
FBS: Fetal bovine serum
FDR: False discovery rate
GEO: Gene expression omnibus
GOBP: Gene ontology biological process
GSEA: Gene set enrichment analysis
GSH: Glutathione
KEGG: Kyoto encyclopedia of genes and genomes
MCL: Markov clustering
MMPs: Matrix metalloproteinases
M-MDSC: Monocyte-like myeloid-derived suppressor cells
MAPK: Mitogen-activated protein kinase
NLS: Nuclear location sequence
PCA: Principal component analysis
PCR: Polymerase chain reaction
PCa: Prostate cancer
PTEN: Phosphatase and tensin homolog
RT-PCR: Reverse transcription-polymerase chain reaction
TANs: Tumor-associated neutrophils
TAMs: Tumor-associated macrophages
UMAP: Uniform Manifold Approximation and Projection
