# Supplementary material for: Myeloid-like tumor hybrid cells in bone marrow promote progression of prostate cancer bone metastasis
Source: J Hematol Oncol. 2023 May 3;16:46. doi: 10.1186/s13045-023-01442-4 (PMC10155318; doi:10.1186/s13045-023-01442-4)
Supplement: Supplementary file 2 — Additional file 2: Figure S1. Comparison of myeloid-like disseminated cancer cells with other disseminated cancer cells. a Heatmap showing the top genes characterized in each cluster. b Heatmap showing the clustered enriched GO BP terms in the differential expressed genes in the cluster 6 versus cluster 3 differential expression analysis. c, d Dot plot showing GO enrichment analysisand KEGG enrichment analysisof differentially expressed genes between cluster 6and cluster 3. Figure S2. The tumor hybrid cells from bone marrow. a Representative bioluminescence image of mouse with bone metastasis after inoculation with RM1through the caudal artery. b Representative flow cytometry dot plot showing the hybrid cells in bone marrow from mice with bone metastasis. c Volcano plot showing the differentially expressed proteins between parental RM1 and hybrid tumor cells. d Enrichment analysis of up-regulated genes in PaGenBase performed by Metascape. Figure S3. Multi-omic analysis of RM1 cells and tumor hybrid cells. a, b Dot plot showing KEGG enrichment analysis of differentially expressed genesor proteins between tumor hybrid cells and RM1 cells. c–e Heatmap showing the clustered enriched GO BP terms in transcriptome analysis, peoteome analysis, and phosphoproteome analysis. Figure S4. The change of metabolism in tumor hybrid cells. a Dot plot showing the metabolic pathways in KEGG enrichment analysis of differentially expressed genes between tumor hybrid cells and RM1 cells. b Bar plot showing the result of GSEA of metabolic pathways in tumor hybrid cells compared with parental RM1 cells. c OPLS-DA scores plot of RM1 cells and tumor hybrid cells. d Validation of OPLS-DA model. e Classification of differential metabolites between tumor hybrid cells and RM1 cells. f Bar plot showing the classification of up-regulated metabolites and down-regulated metabolites in tumor hybrid cells. g Dot plot showing KEGG enrichment of differential metabolites between hybrid tumor cells and pa [file 13045_2023_1442_MOESM2_ESM.docx]

**Myeloid-like Tumor Hybrid Cells In Bone Marrow Promote Progression of Prostate Cancer Bone Metastasis**

Xinyu Ye^1,2^, Xin Huang^1,2^, Xing Fu^1^, Xiao Zhang^1^, Risheng Lin^1^, Wen Zhang^1^, Jian Zhang^1^, Yi Lu^1^

1. School of Medicine, Southern University of Science and Technology, Shenzhen, 518055, China

2. These authors contributed equally: Xinyu Ye, Xin Huang

**Corresponding Author:** Yi Lu and Jian Zhang, School of Medicine, Southern University of Science and Technology, No. 1088 Xue Yuan Blvd, Shenzhen, 518055 Guangdong, China. Email: luy3@sustech.edu.cn (YL), zhangjian@sustech.edu.cn (JZ)


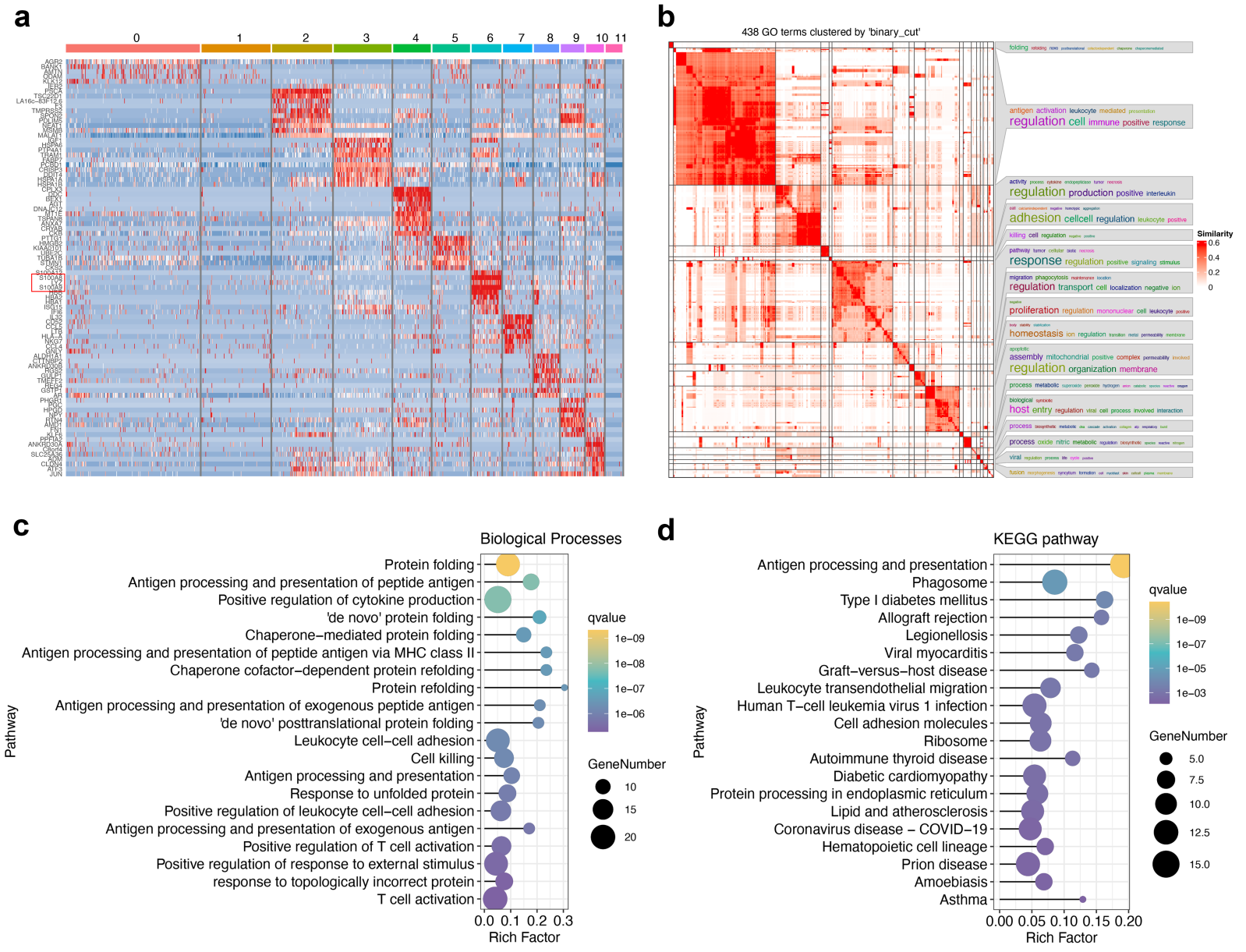


**Additional file 1: Fig. S1 Comparison of myeloid-like disseminated cancer cells with other disseminated cancer cells . a.** Heatmap showing the top genes characterized in each cluster. **b.** Heatmap showing the clustered enriched GO BP terms in the differential expressed genes in the *cluster 6* versus *cluster 3* differential expression analysis. **c, d** Dot plot showing GO enrichment analysis (c) and KEGG enrichment analysis (d) of differentially expressed genes between cluster 6 (myeloid-like tumor cells) and cluster 3.


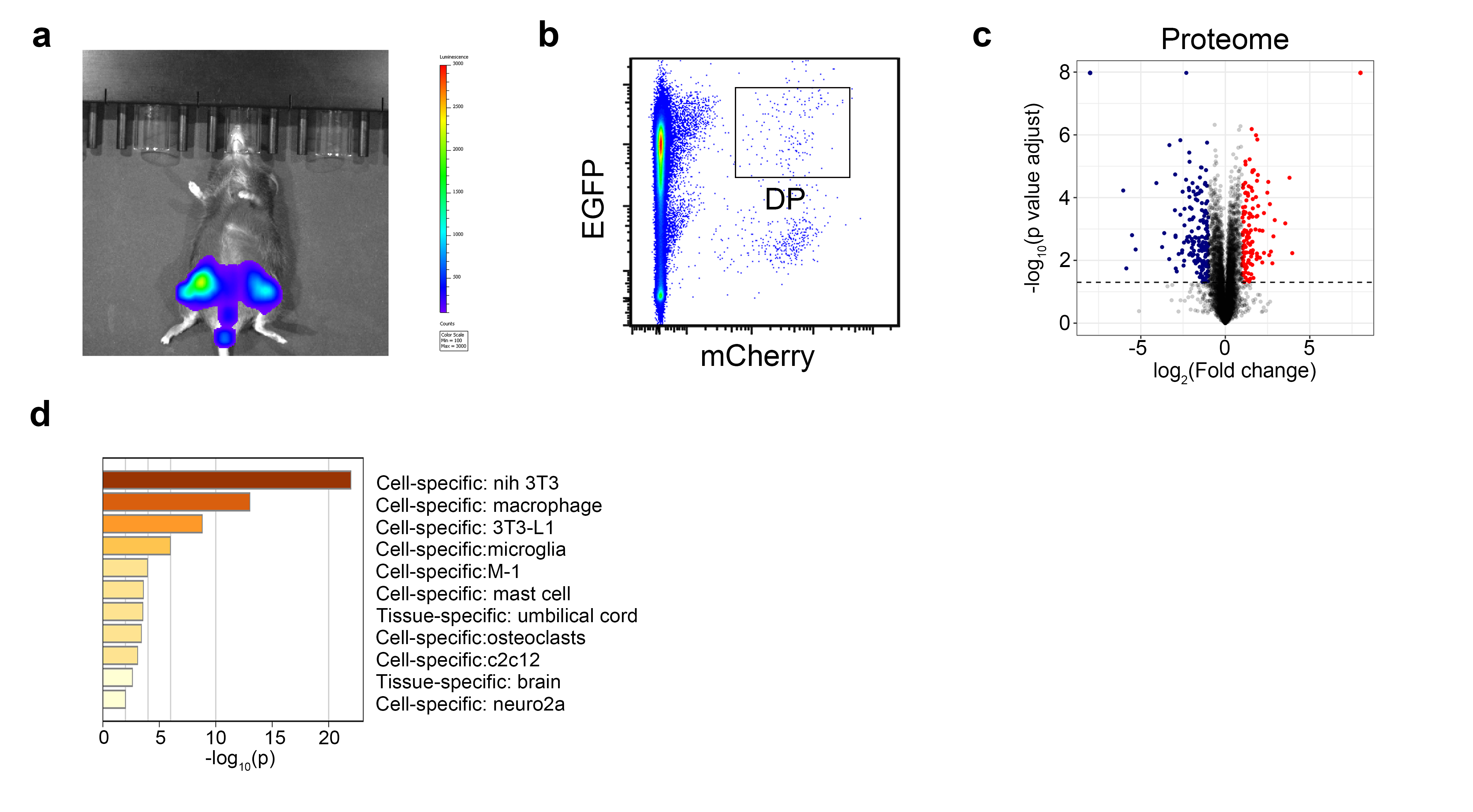


**Additional file 2: Fig. S2 The tumor hybrid cells from bone marrow. a.** Representative bioluminescence image of mouse with bone metastasis after inoculation with RM1 (luciferase) through the caudal artery. **b.** Representative flow cytometry dot plot showing the hybrid cells in bone marrow from mice with bone metastasis. **c.** Volcano plot showing the differentially expressed proteins between parental RM1 and hybrid tumor cells. **d.** Enrichment analysis of up-regulated genes in PaGenBase performed by Metascape.


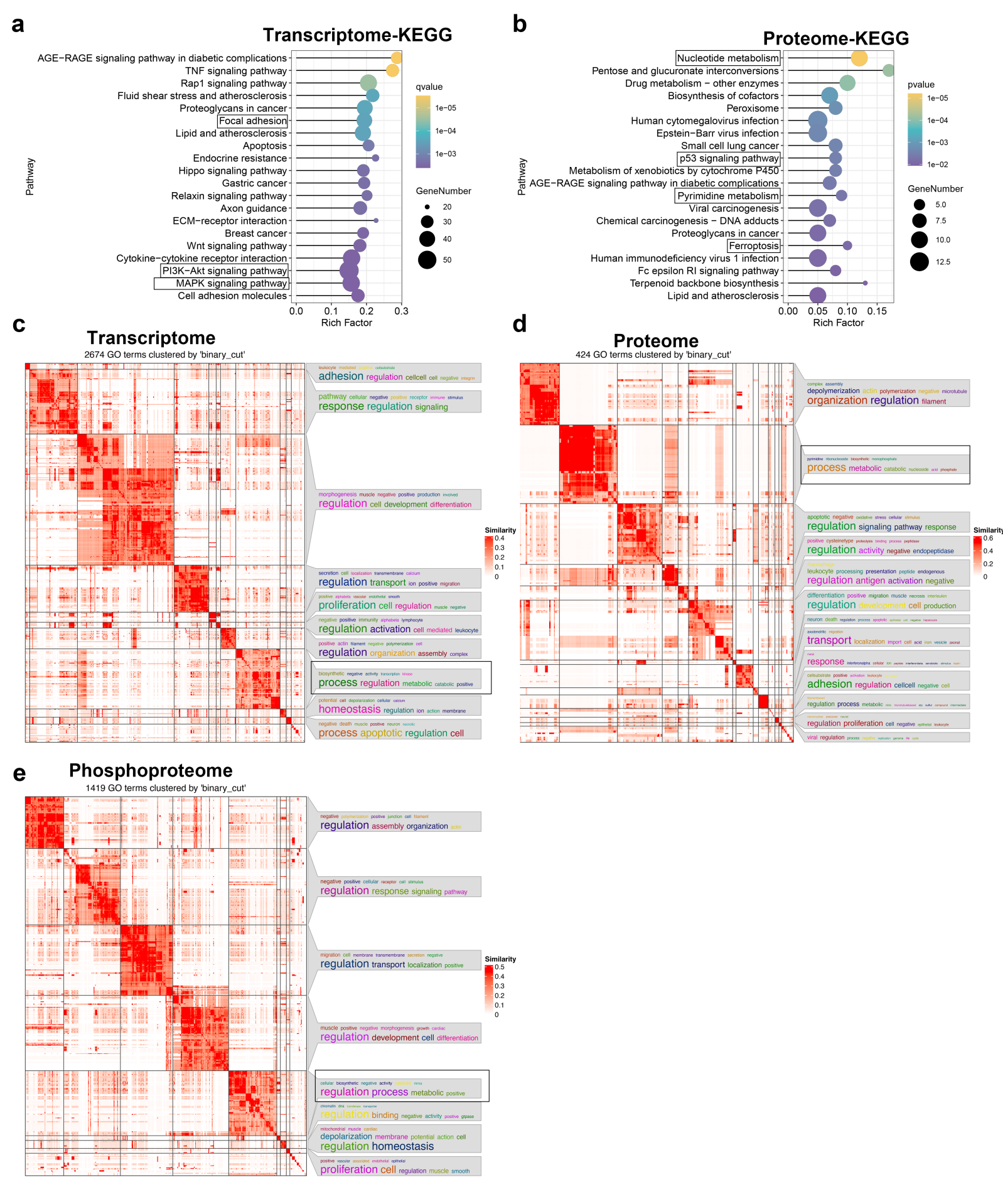


**Additional file 3: Fig. S3 Multi-omic analysis of RM1 cells and tumor hybrid cells. a,b** Dot plot showing KEGG enrichment analysis of differentially expressed genes (a) or proteins (b) between tumor hybrid cells and RM1 cells. **c-e,** Heatmap showing the clustered enriched GO BP terms in transcriptome analysis (c), peoteome analysis (d), and phosphoproteome analysis (e).


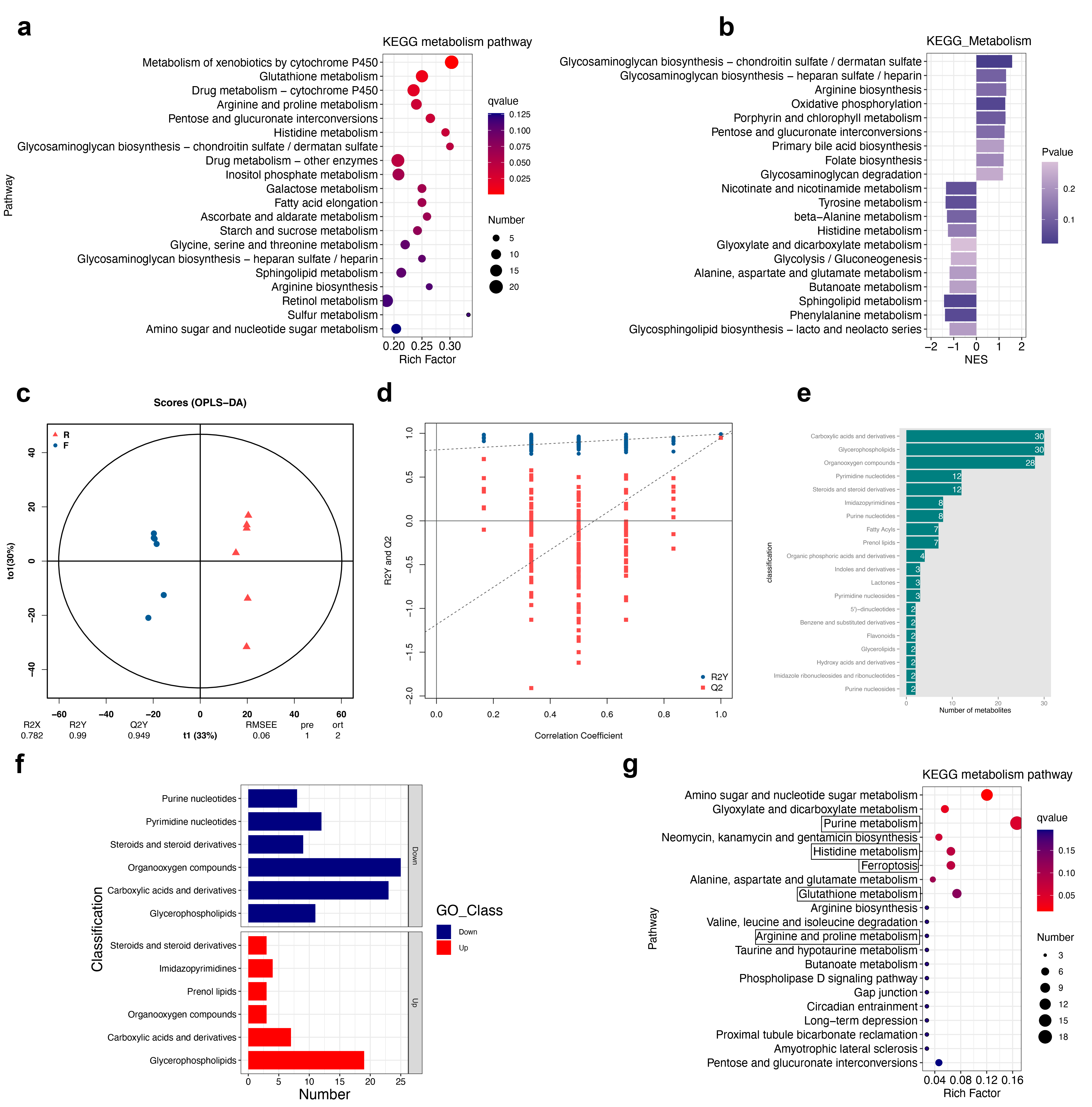


**Additional file 4: Fig. S4 The change of metabolism in tumor hybrid cells. a.** Dot plot showing the metabolic pathways in KEGG enrichment analysis of differentially expressed genes between tumor hybrid cells and RM1 cells. **b.** Bar plot showing the result of GSEA of metabolic pathways in tumor hybrid cells compared with parental RM1 cells. **c.** OPLS-DA scores plot of RM1 cells and tumor hybrid cells (n=6). **d.** Validation of OPLS-DA model. **e.** Classification of differential metabolites between tumor hybrid cells and RM1 cells. **f.** Bar plot showing the classification of up-regulated metabolites and down-regulated metabolites in tumor hybrid cells. **g.** Dot plot showing KEGG enrichment of differential metabolites between hybrid tumor cells and parental RM1 cells.


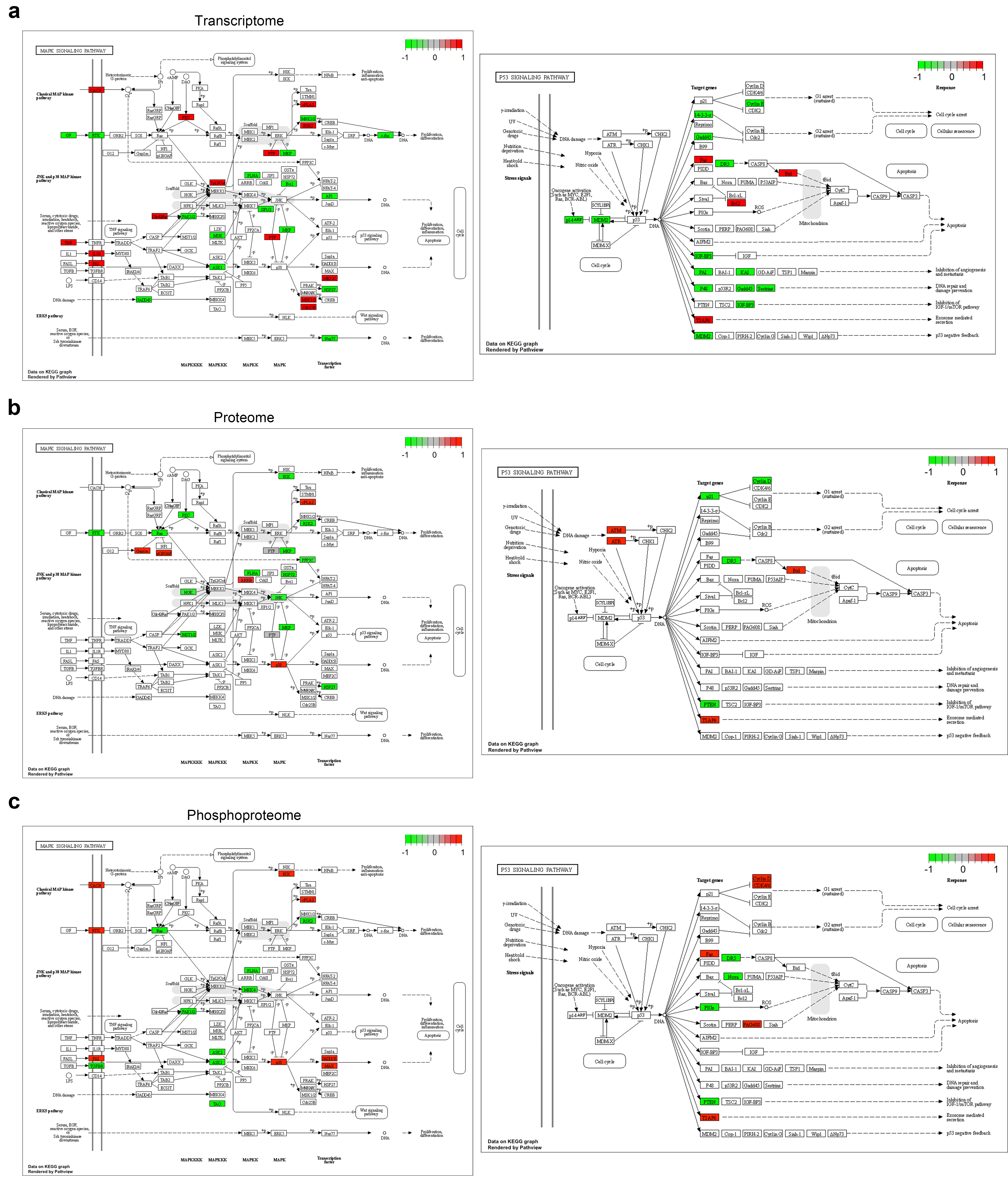


**Additional file 5: Fig. S5** **The change in MAPK signaling pathway and p53 signaling pathway.** The differentially expressed genes (a), proteins (b), or proteins with different phosphorylation statuses (c) in MAPK signaling pathway and p53 signaling pathway.


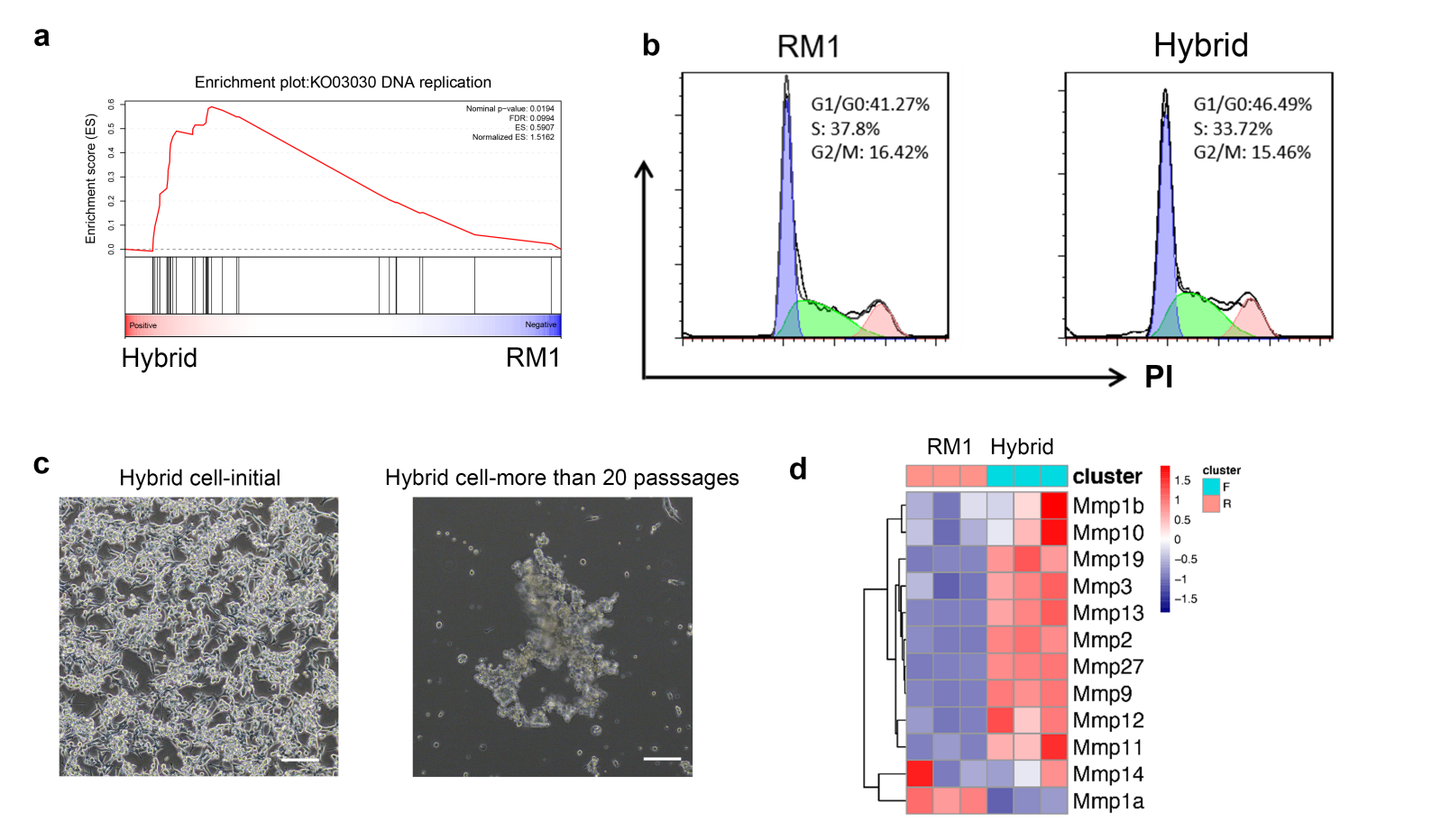


**Additional file 6: Fig. S6 The change of DNA replication and cell adhesion in tumor hybrid cells. a.** GSEA of DNA replication pathways in tumor hybrid cells compared RM1 cells. **b.** Cell cycle analysis of RM1 and tumor hybrid cells. **c.** The morphological change of tumor hybrid cells after long term cultured *in vitro* (scale bar:100 µm), most hybrid cells became suspension growth. **d.** The differentially expressed MMPs family genes between RM1 cells and hybrid cells.


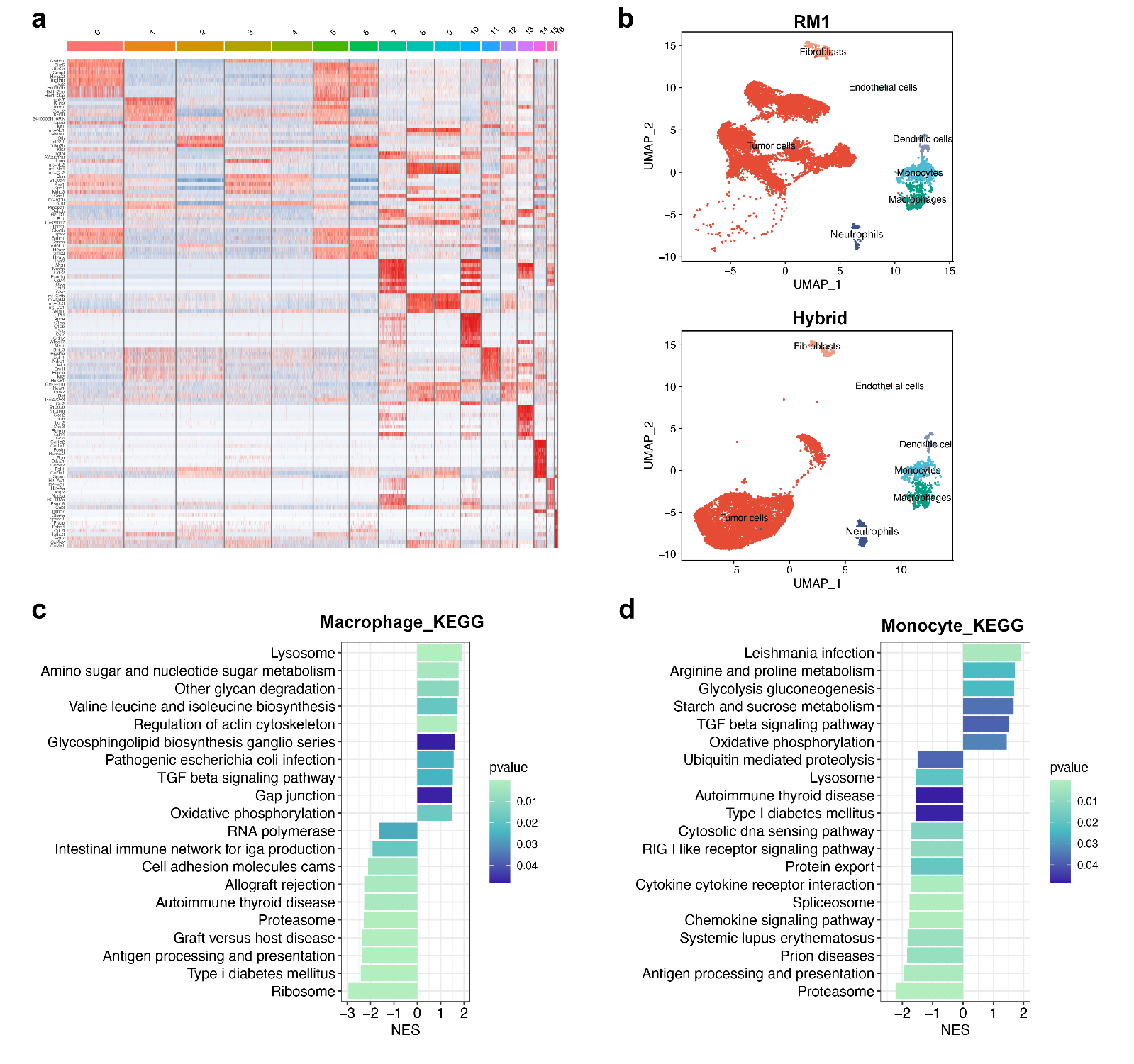


**Additional file 7: Fig. S7 Single-cell RNA sequencing of RM1-derived tumor cells and hybrid cell-derived tumor cells. a.** Heatmap showing the top genes characterized in each cluster. **b.** UMAP plots showing the clusters colored by cell type in RM1-drived tumor (upper) and hybrid cell-derived tumor (lower). **c, d** Bar plots showing the results of GSEA of KEGG pathways in macrophages (c) and monocytes (d) from hybrid cell-derived tumors compared with those from RM1-derived tumors.


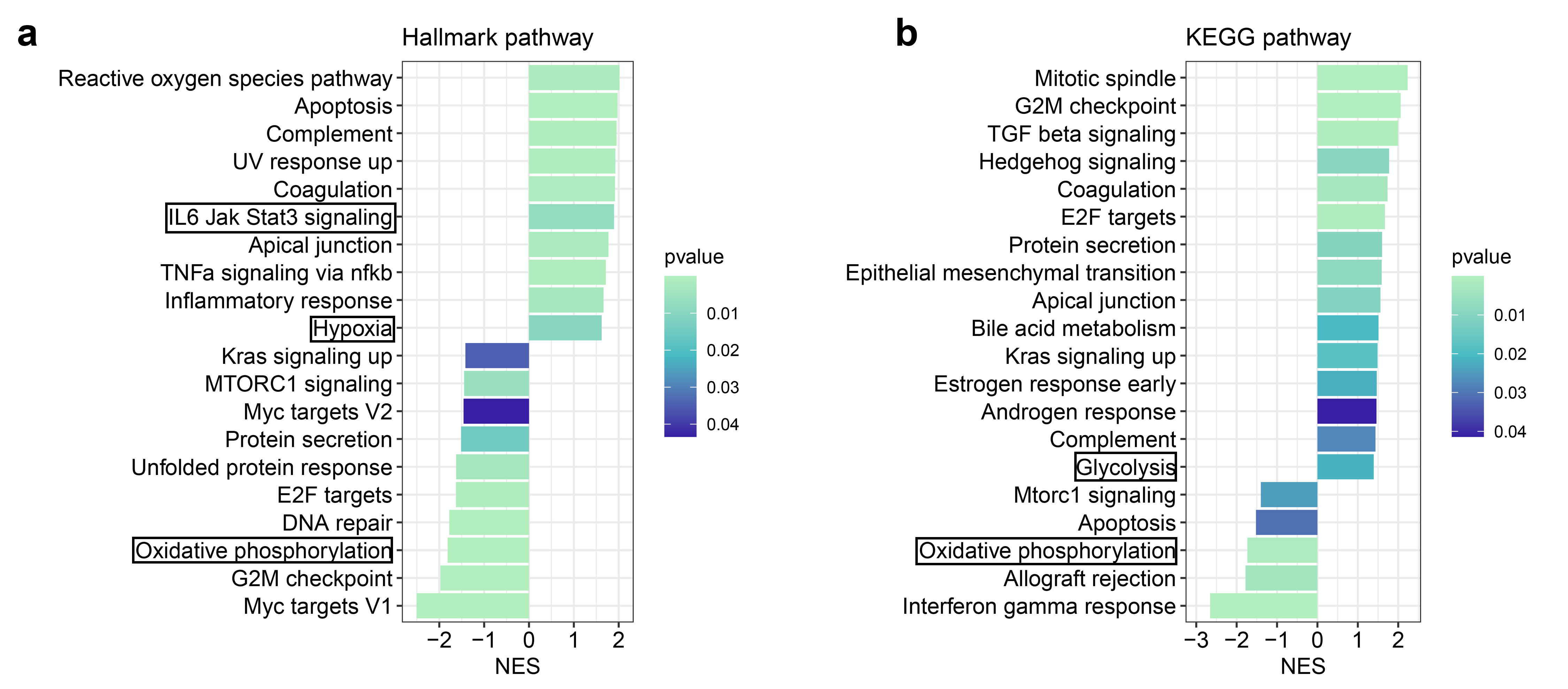


**Additional file 8: Fig. S8** Bar plot showing the results of GSEA analysis of hallmark pathway (a) and KEGG pathway (b) in neutrophils from hybrid cell group compared those from RM1 group.


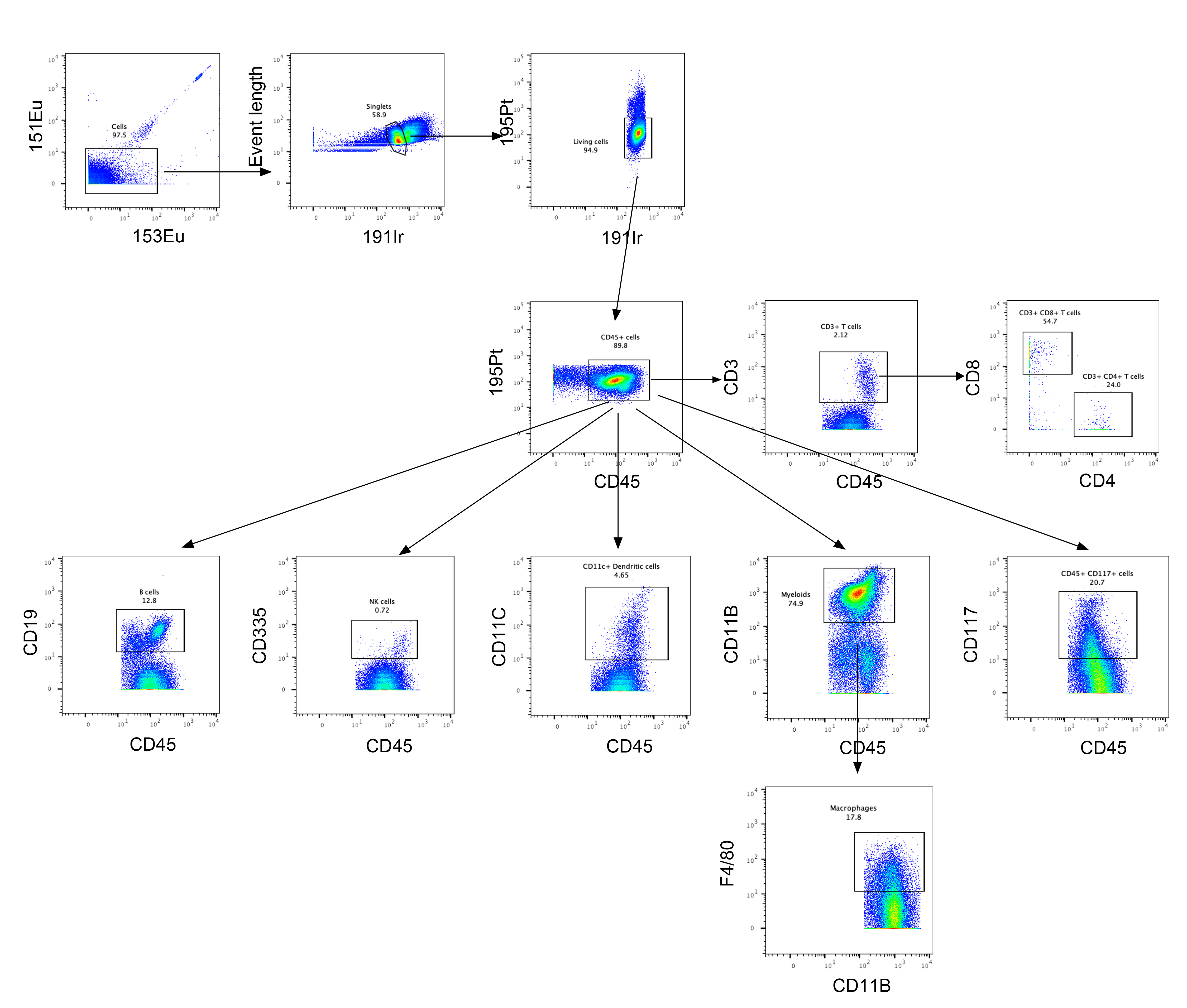


**Additional file 9: Fig. S9 Gating strategy for each cluster of cells in the bone marrow from normal mice, mice with RM1-derived bone metastasis, and mice with hybrid cell-derived bone metastasis.**


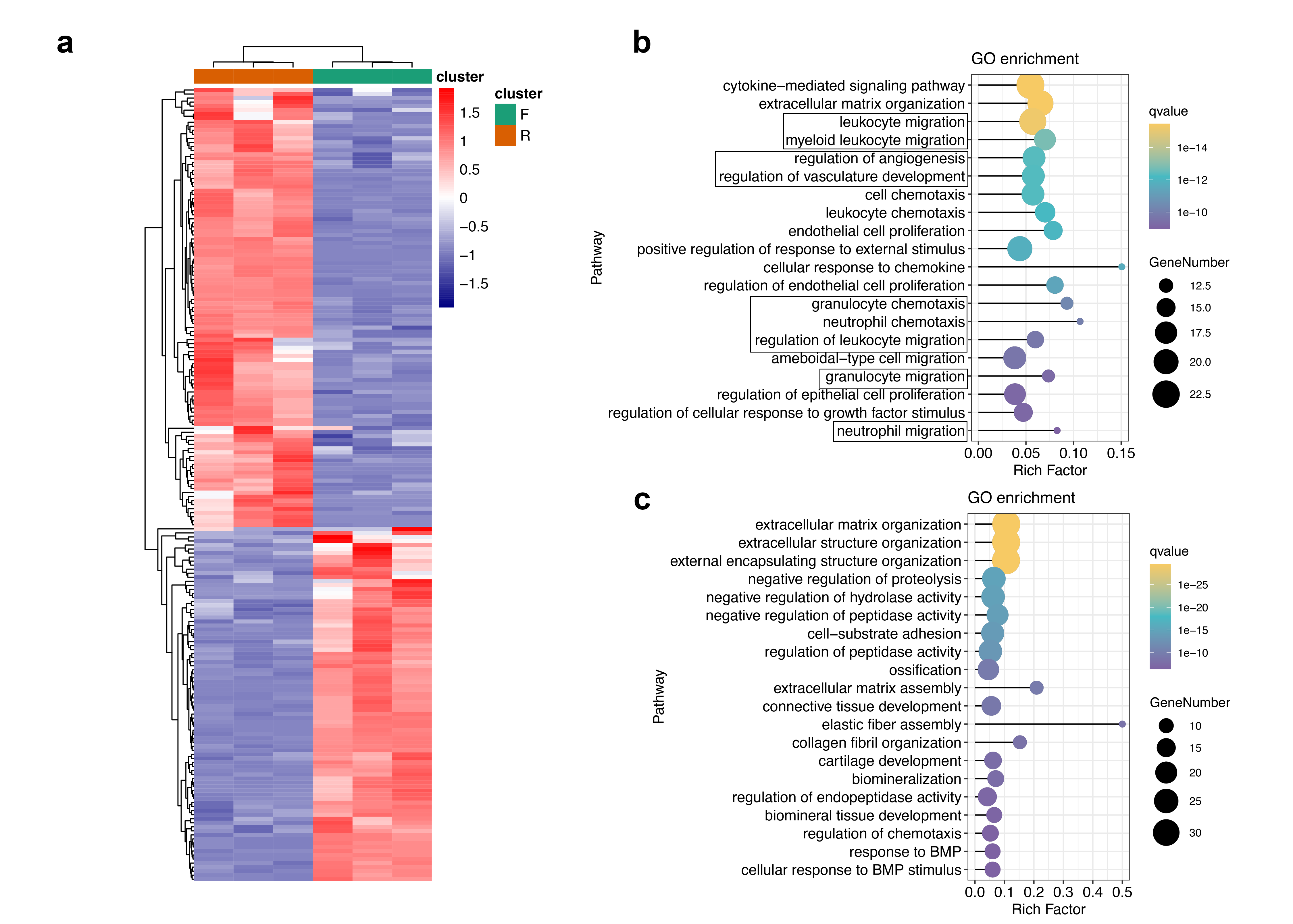


**Additional file 10: Fig. S10 the secretome analysis of RM1 and hybrid tumor cells.** a. Heatmap showing the differentially expressed chemokines and cytokines between parental RM1 and hybrid tumor cells. b. c GO enrichment analysis of up-regulated chemokines and cytokines (a) or downregulated chemokines and cytokines (b) in tumor hybrid cells.

**
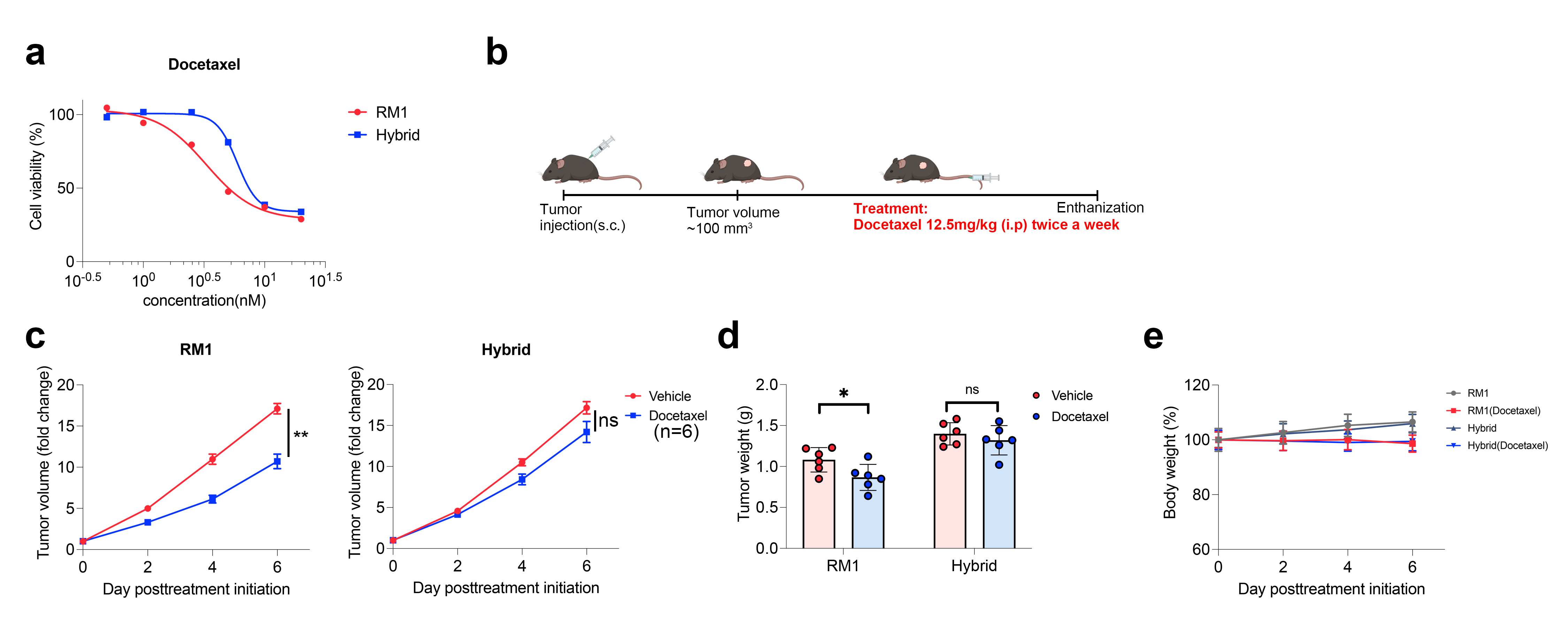
**

**Additional file 11: Fig. S11 Tumor hybrid cells are resistant to docetaxel *in vitro* and *in vivo*. a.** Viability of RM1 and hybrid cells after docetaxel treatment. **b.** Schematic illustration of the experimental design using docetaxel treatment. **c.** The growth curve of RM1 tumor or hybrid tumors after treatment with docetaxel (n=6). **d.** Tumor weight measured at the endpoint of the experiment after treatment with docetaxel (n=6). **e.** The growth curve of body weight in mice treated with docetaxel or vehicle (n=6). ns: not significant, *P < 0.05, **P < 0.01, ***P < 0.001.

**
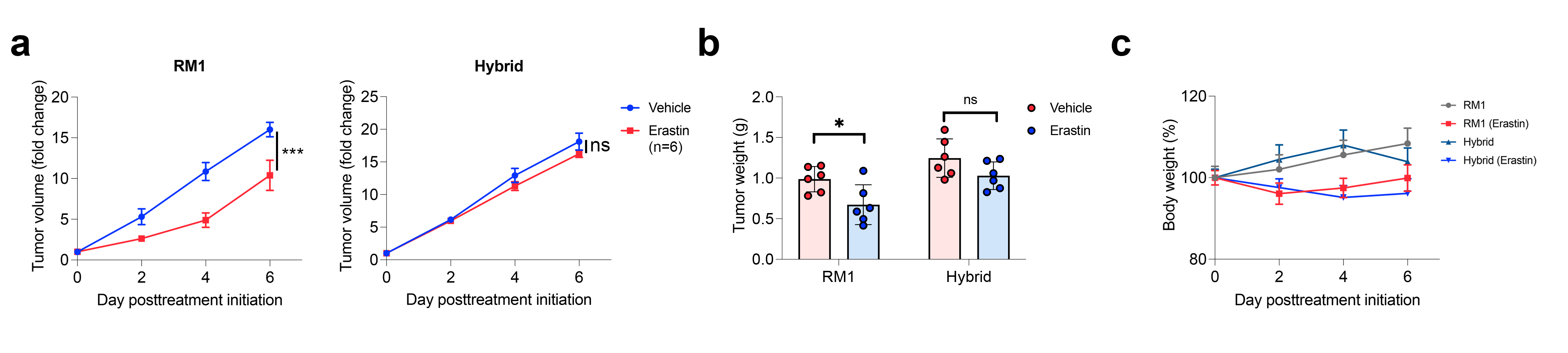
**

**Additional file 12: Fig. S12 Tumor hybrid cells are resistant to erastin *in vivo*. a.** The growth curve of RM1 tumor or hybrid tumors after treatment with erastin (n=6). **b.** Tumor weight measured at the endpoint of the experiment after treatment with erastin (n=6). **c.** The growth curve of body weight in mice treated with erastin or vehicle (n=6). ns: not significant, *P < 0.05, **P < 0.01, ***P < 0.001.
